# Enantioselectivity of Isomerization of *n*-Humulone to *trans*- and *cis*-*n*-Isohumulones

**DOI:** 10.3390/molecules31152731

**Published:** 2026-08-06

**Authors:** Bruce C. Hamper, Gregory Giovine, Rajamoni Jagan, Trevor Smith

**Affiliations:** Department of Chemistry & Biochemistry, University of Missouri—St. Louis, 43 Benton Ct., St. Louis, MO 63121, USA; gmg9ng@umsl.edu (G.G.); jrbrm@umsl.edu (R.J.); ts7gf@umsl.edu (T.S.)

**Keywords:** humulone, isohumulone, photochemistry, continuous flow synthesis, chiral HPLC, precision photochemistry, oxadi-π-methane rearrangement, ODPM

## Abstract

The enantiomeric composition of products obtained from isomerization of *n*-humulone **1a** to *trans*- and *cis*-*n*-isohumulones **2a** and **3a**, respectively, was determined by chiral HPLC analysis of the isolated *trans* and *cis* isomers. Enantiomers of humulone **1** were readily resolved on a Whelk-O1 chiral stationary phase (CSP), whereas the isomeric isohumulones **2** and **3** were separated on a carbohydrate-based IC-3 CSP. Magnesium-catalyzed isomerization of *n*-humulone under basic conditions gave mixtures of *trans*- and *cis*-*n*-isohumulones exhibiting an increasing preference for the cis isomer at lower temperatures. At either 10 °C in CH_2_Cl_2_/H_2_O or reflux in methanol-water, the *trans*-(−) (4*S*,5*S*)-**2a** isomer was obtained in greater than 95% ee, while diastereomeric *cis*-**3a** exhibited 88% ee of the (+)-(4*S*,5*R*) enantiomer. Precision continuous flow photochemical isomerization using 395 nm LEDs provided *trans*-(−)-**2a** in greater than 95% ee in an isolated yield of 93%. However, photochemical isomerization with 365 nm LEDs gave a mixture of isomers consisting of 46% ee for the *trans*-(−)-**2a** and greater than 95% ee for the *cis*-(−)-(4*R*,5*S*)-**3a** enantiomer. The photochemically obtained (−)-**3a** isomer has the opposite absolute configuration compared to thermal isomerization. An oxadi-π-methane rearrangement is proposed to account for the formation of isohumulone isomers under photochemical conditions.

## 1. Introduction

The hops plant (*Humulus lupulus* L.) is primarily known for the use of the flowering inflorescence or strobile as one of the major components, along with malts, water and yeast, in the production of beer [1,2,3,4,5]. Hops in brewing give desirable bitter and aroma flavors to the final product and have the added benefit of acting as a preservative, helping prevent infection by undesirable bacteria such as lactobacillus or pediococcus [6,7,8]. Recently, hops have found use as a flavoring agent and desirable component of non-alcoholic beverages such as hop teas and relaxation drinks [9,10,11]. Perhaps less known is the use of hop extracts for over-the-counter nutritional supplements purporting a wide range of potential benefits including treatment of anxiety, insomnia, symptoms of menopause, inflammation and an extended list of other maladies [12,13,14]. Hops extracts have been evaluated for the alleviation of early menopausal symptoms in randomized placebo-controlled trials [15]. In a recent clinical trial, hop extracts have been shown to modulate gut peptide hormone secretion in healthy-weight men [16].

**Figure 1:**
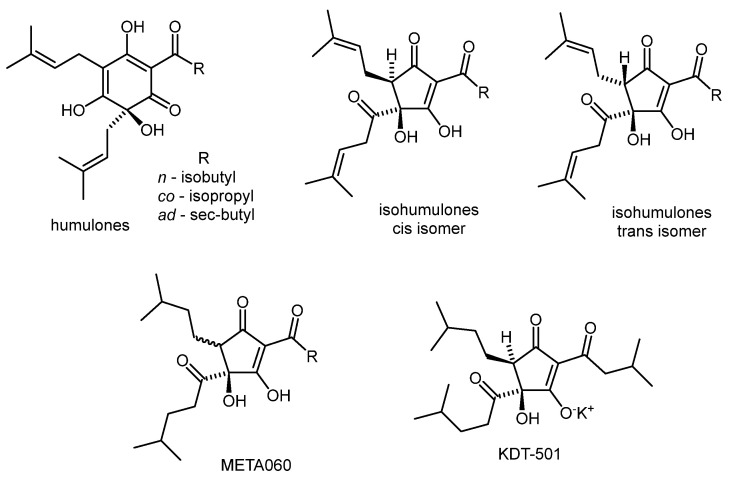
Humulones and derivatives obtained from *Humulus lupulus* L. (hops).

Hops have also proven to be an excellent source of isolated biologically active compounds [17,18,19] (Figure 1). The harvested strobiles of the hops plant contain between 2 and 15% by weight of humulones, a mixture of prenylated oxidized phloroglucinols [20]. The major homologs of humulone differ in the alkyl ketone substitution of the cyclohexadienone ring, with over 95% of the content represented by isobutyl (*n*-), isopropyl (*co*-) and sec-butyl (*ad*-) ketones. The homolog *n*-humulone has been evaluated for sleep-promoting activity [21] and shown to inhibit cell growth of lung and bone cancer cells [22]. Humulones can be isomerized to isohumulones by both thermal and photochemical methods [23]. Thermal isomerization is frequently employed and results primarily in six products consisting of a mixture of *cis* and *trans* isomers of each of the three most prominent homologs (*n*-, *co*- and *ad*-isohumulones). Following standard convention, the *cis* isomer indicates a *cis* arrangement between the C4 hydroxyl group and the C5 isoprenyl group. Isohumulone mixtures have been evaluated for medical nutrition therapy to treat or prevent metabolic syndrome and related disorders such as diabetes, inflammation and impaired glucose tolerance [24]. In a study with pre-diabetic patients, daily administration of capsules containing 48 mg of isohumulones over a 12-week period led to a significant reduction in body weight and BMI [25]. The brewing industry has developed reduced isohumulones for providing light-stable bittering agents for beer [26]. Unlike isohumulones, which are readily oxidized and sensitive to light, the reduced isohumulones have improved stability and provide bitter flavors that do not degrade in the final beverage [27]. META060 is a reduced tetrahydro-isohumulone product that consists of a mixture of the *cis* and *trans* isomers of the *n*-, *co*- and *ad*-homologs. Notably, a single oral dose of 940 mg of META060 in human trials was found to provide plasma levels sufficient to inhibit LPS-stimulation of TNFα and IL-6, suggesting its potential for treatment of chronic inflammatory disease [28]. Further development of reduced isohumulone derivatives led to the identification of KDT-501, which has been in phase 2 clinical trials for control of metabolic disease in insulin-resistant patients [29]. KDT-501 is also being considered for control of polycystic ovarian syndrome and non-fatty acid liver disease [30,31]. 

Our interest in the natural products obtained from hops began with the introduction of a special topics course on brewing science for non-science major undergraduate students [32]. During the development of this course, we explored analytical methods for the analysis of humulone and isohumulone in hops and beer [33,34]. Isotopically labeled isohumulones were prepared for use in stable isotope dilution assays and potential analysis of degradation products [35]. A continuous flow photochemical method was developed for stereoselective formation of gram quantities of *trans*-isohumulones in nearly quantitative yield [36]. A surprising result of the photochemical investigation was the impact of light-emitting diode (LED) wavelength on the product distribution of isohumulones obtained from *n*-humulone. The advent of LEDs and monochromatic lasers in synthetic photochemistry has resulted in exquisite control of reaction conditions and has been termed ‘precision photochemistry’ [37]. 

To further our understanding of humulone to isohumulone isomerization, we needed to obtain racemic samples of these compounds and develop chiral HPLC methods for enantiomeric resolution. In addition to allowing greater insight into specific reactions, these methods can greatly advance our evaluation of compounds for biological evaluation. The enantiomeric purity of humulones and derivatives present in beverages and evaluated in biological studies is only infrequently considered. In this report, we investigate the resolution and enantiomeric purity of isohumulones obtained from both thermal and photochemical processes and provide methods for stereochemical evaluation of humulone-derived products.

## 2. Results

### 2.1. Isomerization of Humulones

**Scheme 1:**
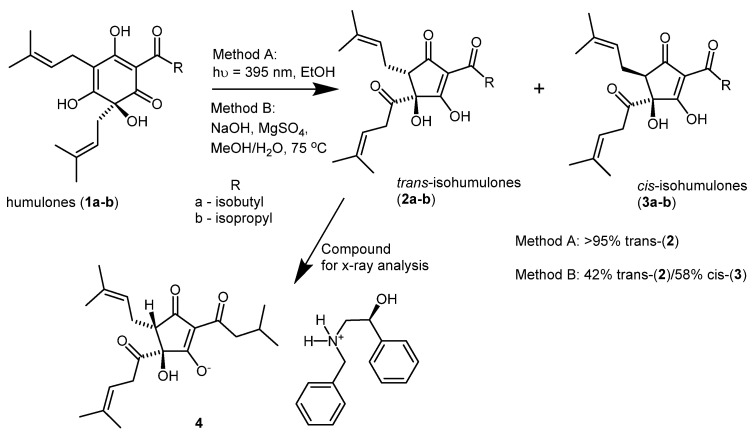
Isomerization of humulones **1** to diastereomeric *trans*/*cis*-isohumulones **2** and **3**. Compound **4**, the chiral amine salt of (−)-**2a**, was prepared for X-ray analysis.

In 1965, Clarke and Hildebrand reported the isolation of *trans*-*n*-isohumulone **2a** from photochemical isomerization of *n*-humulone **1a** [38]. Their crystalline product had a specific rotation of −7.6° (methanol) and stereochemistry consistent with the isolated *trans* product **2a** (−) obtained by base-catalyzed isomerization of **1a**. In our studies, the *trans* isomer **2a** obtained by continuous flow photochemistry had a specific rotation of -9^0^ (ethanol) [36], which, within experimental error and slight solvent differences, agrees with previous reports [39]. Considering the modest specific rotation of this compound, it would be difficult to ascertain the enantiomeric purity from rotation data alone. Thermal or base-catalyzed isomerization results in a mixture of *cis* and *trans* isomers, in which the optical purity of the isomers also indicates remarkable enantioselectivity resulting in the *trans*-**2a** (−) and *cis*-**3a** (+) enantiomers (Scheme 1) [23,35]. Based on X-ray crystallographic data, the absolute configuration has been assigned as 4*S*,5*S* for (−)-**2a** and 4*S*,5*R* for (+) **3a** [40]. Although these are considered enantiomerically pure products of these reactions, it has been suggested that as much as 15% racemization might occur in the thermal process [41].

### 2.2. Chiral Resolution of Enantiomeric Humulones ***1*** and Isohumulones ***2*** and ***3***

**Figure 2:**
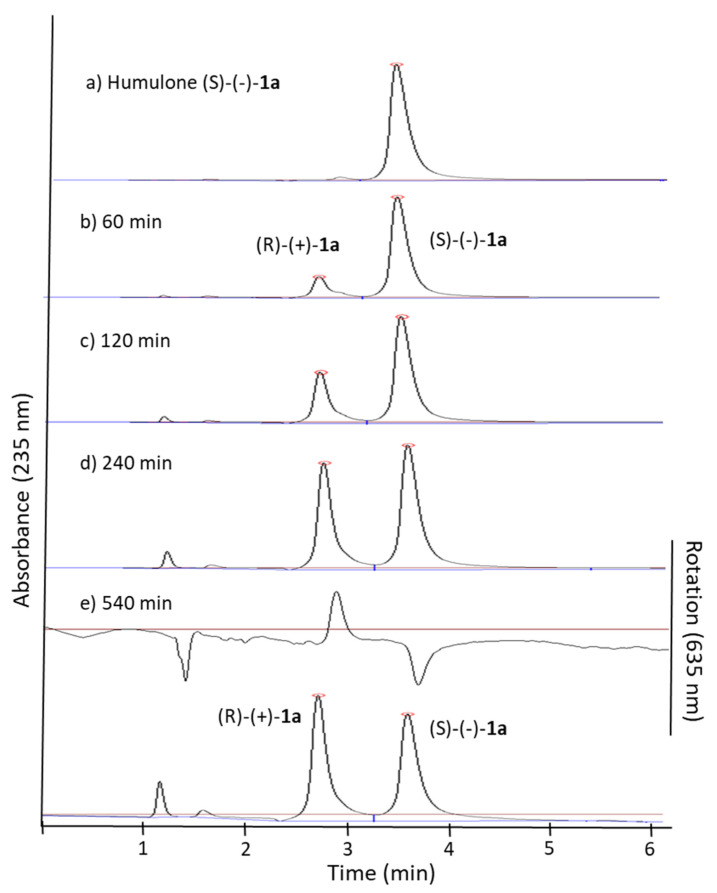
HPLC resolution of humulone **1a** enantiomers during thermal racemization. Pirkle Covalent (*R*,*R*)-WhelkO1 chiral stationary phase; 2% isopropanol/hexane/0.1% TFA mobile phase. The final product (chromatogram e) was analyzed by detection at both UV 235 nm and polarimetric response at 635 nm. The (*R*)-(+)-1a peak shows a positive peak indication (+) rotation, whereas the (*S*)-(−)-1a exhibits a negative peak (see Supplementary Material for details).

**Figure 3:**
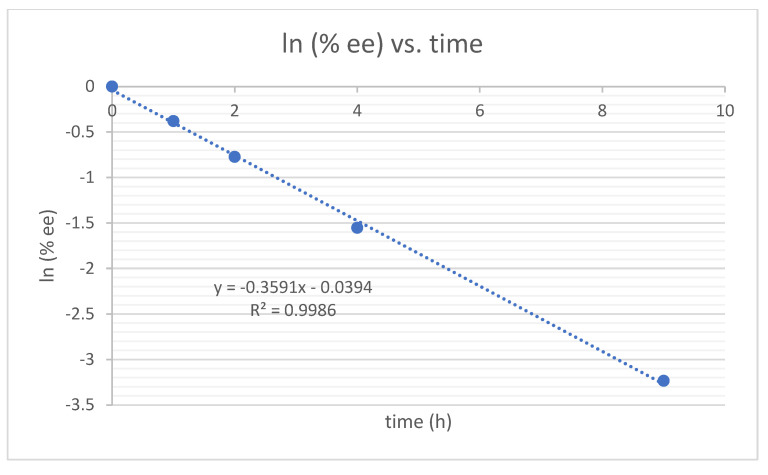
Changes in ln of the percent enantiomer excess (% ee) vs. time for the racemization of **1a** in isooctane at 130 °C. Linear regression of the five experimental data points gave a rate constant of 0.359/h corresponding to a half-life of 116 min.

To evaluate the enantioselectivity of photochemical isomerization of **1** to **2**, we prepared authentic samples of racemic **1a** and **1b**. Racemization of **1a** was first reported by Anteunis and Verzele [42] and has been achieved in various non-polar solvents at elevated temperatures [22,23,40]. We found it convenient to conduct the racemization in isooctane under an argon atmosphere in the pressure tube at 130 °C. The course of the reaction was determined by chiral HPLC analysis of aliquots taken at various time points. Enantiomers of **1a** were readily separated by chiral HPLC using a (*R*,*R*)-Whelk-O1 chiral stationary phase (Figure 2) [43]. Humulones are weak acids with a reported pKa of 5.0 [44]. As a result, an acid modifier was required in the chromatographic analysis to obtain reasonable peak shape. Isocratic conditions of 2% isopropyl alcohol in hexanes with 0.1% trifluoroacetic acid provided baseline separation of the enantiomers of **1a**. Nearly complete racemization was achieved in 9 h with the unnatural *R*-(+) enantiomer eluting first. A plot of the natural log of the enantiomeric excess (ln % ee) vs. time in minutes resulted in a straight line indicating first-order kinetics for the racemization process (Figure 3). The rate constant determined from linear regression of this plot is krac = 0.359 h^−1^, which corresponds to a half-life of 116 min.

The homolog *co*-humulone **1b** was racemized by the same process as **1a**. Some decomposition was also observed during the racemization, resulting in lower yields of the desired racemic products (+/−) **1a** and **1b**. The humulones are very sensitive to light and oxidation, resulting in losses during purification and handling. Isolated yields after reverse-phase HPLC purification of **1a** and **1b** were between 36 and 39%.

Photochemical isomerization of racemic (+/−) **1a** and **1b** using a continuous flow apparatus resulted in the expected *trans* isomers **2a** and **2b**. The yield of racemic *trans* **2a** (41.6%) was lower than obtained for (−)-**2a** (93%) from isomerization of the enantiomerically pure humulone (−)-**1a**. Racemic **2a** was obtained as a semi-solid and required additional recrystallization to obtain analytically pure material. The enantiomers were not separable using the Whelk-O1 CSP. Chromatographic resolution was investigated on several available CSPs to find a suitable alternative (Table 1). Resolution of the parent humulone **1a** was readily achievable on a variety of carbohydrate-based CSPs (IA3, IB3, IC3, ADH) in addition to the Whelk-O1 bonded phase. The IC3 CSP provided an improvement in separation factor α of 2.85; however, peak resolution was significantly lower due to the lower resolution performance of the column. In fact, all the carbohydrate CSPs had lower resolving power and fewer observed theoretical plates compared to the Whelk-O1 bonded phase. Surprisingly, the separation of cohumulone **1b** on either the Whelko-1A or the IC3 (α = 1.11 and 1.25, respectively) was significantly less than observed for **1a**. For all these cases, the unnatural (+) enantiomer of humulones **1** was eluted first from the CSP. We did not observe resolution of the isohumulones **2** or **3** on the Whelko-1A CSP. However, we were able to obtain greater-than-baseline resolution of enantiomers of *trans*-isohumulones **2a** and **2b** on the IC3 CSP with the (+) enantiomer eluting first.

The racemic *cis*-isohumulones **3a** and **3b** were obtained by magnesium-catalyzed thermal isomerization at 10 °C [45]. Under these conditions, a mixture of the *cis* and *trans* isomers is obtained in an 83:17 ratio. The minor *trans* isomer can be removed by selective fractional crystallization with dicyclohexylamine. Separation factors for the *cis* isomers were less than the values observed for the *trans* isomers. However, separation of the purified *cis*-isohumulones, **3a** and **3b**, on an IC3 CSP provided sufficient resolution with the (−) enantiomers eluting first.

### 2.3. Enantio- and Diastereoselectivity of Isomerization of Humulone ***1a*** to Isohumulones ***2a*** and ***3a***

With the analytical chiral chromatography method in hand, we turned our attention to analysis of isomerization of naturally occurring (*S*)-(−)-humulone **1a** to the *trans*- and *cis*-isohumulones **2a** and **3a** (Table 2). While slight changes in reaction conditions can led to changes in enantiomeric composition, the examples provided are instructive for demonstrating the value of chiral HPLC analysis. IC3 CSP was effective for the separation of the isohumulone enantiomers; it did not provide separation of the *cis* and *trans* diastereomers. The *trans-cis* ratios of products from the isomerization reactions were determined by comparison of ^1^H NMR and reverse-phase C18 HPLC with authentic samples. Magnesium-catalyzed isomerization under basic conditions afforded mixtures of the *cis* and *trans* isomers in which the *cis* isomer was the major component. The reaction at 10 °C required 5 days for completion and gave an 83:17 mixture of **3a** and **2a**, respectively. At reflux in methanol-water, the reaction gave a 57:43 mixture of isomers. In both cases, we observed enantiospecific formation of the *trans* isomers in greater than 95% ee as determined by chiral HPLC. Isolation of the *cis* isomer, however, resulted in a mixture of enantiomers having an 88% enantiomer excess of the positive 4S,5R isomer. The observation that the *cis* isomer is obtained as a mixture of enantiomers is not completely understood. However, this led us to consider the potential for partial racemization of isohumulones during food processing, such as encountered in the preparation of beer, hop tea and other products. Acetate buffer conditions at pH 5.5 were investigated to provide a suitable comparison for isomerization encountered during beer brewing [46]. Under these weakly acidic conditions, we obtained a 27:71 mixture of *trans* and *cis* isomers, respectively. Separation of the two diastereomers followed by chiral HPLC analysis indicated stereospecific isomerization in the formation of both (−)-**2a** and (+)-**3a**.

**Figure 4:**
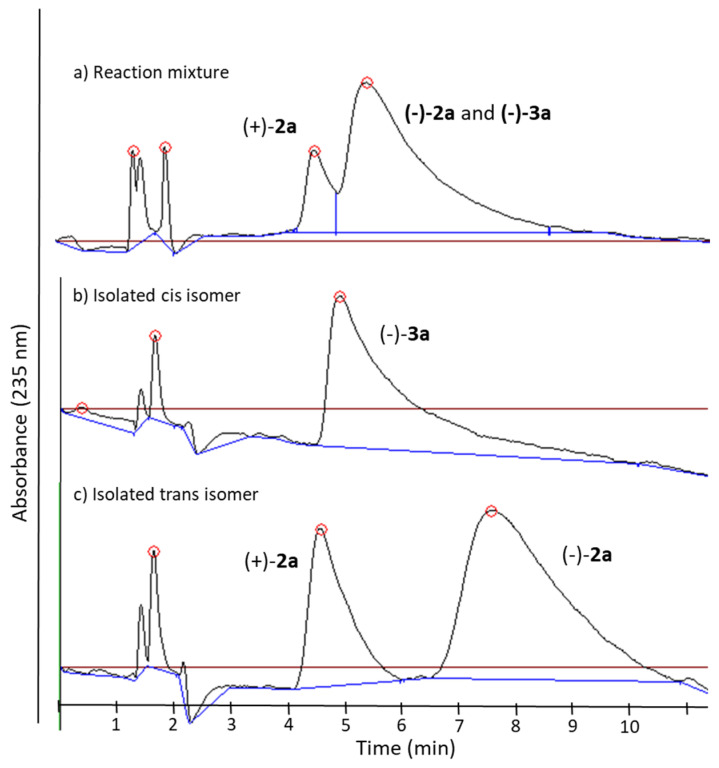
Chiral HPLC resolution of the products obtained from flow photochemical isomerization of **1a** at 365 nm. (a) reaction mixture; (b) isolated *cis* isomer **3a**; (c) isolated *trans* isomer **2a**.

The photochemical continuous flow reaction using a 395 nm LED light source gave stereospecific and enantiospecific formation of the (4*S*,5*S*)-(−)-**2a** product in 97% ee. The photoflow reaction carried out with higher-energy 365 nm LEDs gave significantly different results, providing an 80:20 mixture of *trans*-**2a** and *cis*-**3a**. Analysis of the reaction mixture by chiral HPLC showed two peaks but did not provide separation of the diastereomers (Figure 4a). To determine enantioselectivity of the individual products, it was necessary to separate the *cis* and *trans* diastereomers by semi-preparative reverse-phase C18 chromatography. The purified *cis*-**3a** isomer proved to be at least 95% enantiomerically pure based on the levels of detection provided by HPLC analysis. While thermal isomerization provides enantioselective formation of the (+)-(4*S*,5*S*)-**3a** enantiomer, photochemical isomerization at 365 nm gave stereospecific formation of the opposite enantiomer (−)-(4*R*,5*S*)-**3a** (Figure 4b). Surprisingly, chiral HPLC of the purified *trans* isomer gave a 73:27 mixture of enantiomers, resulting in 46% ee of (−)-**2a** (Figure 4c). The products obtained from both 395 and 365 nm reactions are remarkably stable to reaction conditions. Reintroduction of (−)-**2a** or (+)-**3a** to the continuous flow photoreactor did not lead to any racemization or isomerization of the products.

**Scheme 2:**
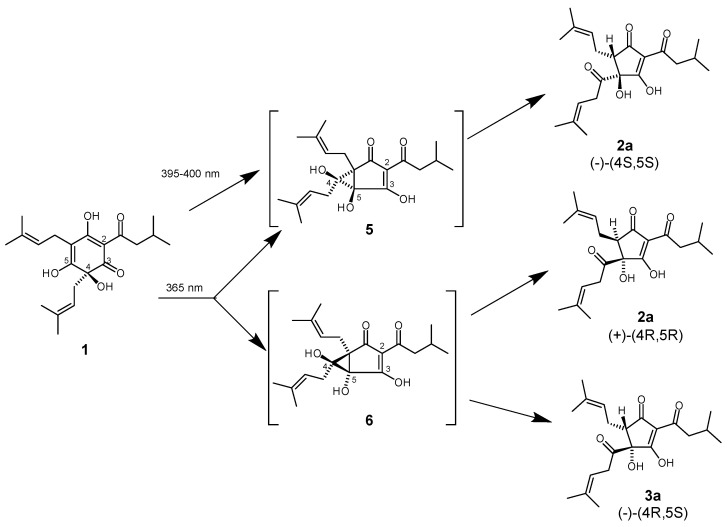
Precision photochemical isomerization of humulone **1a** to isohumulones **2a** and **3a**. Intermediates **5** and **6** are displayed in brackets. Photochemistry at 395–400 nm gave exclusive formation of (−)-**2a**. Photochemical reaction at 365 nm provided a mixture of 58% (−)-**2a**, 22% (+)-**2a** and 20% (−)-**3a**. This can be alternatively described as 80% *trans*-(−)-**2a** (46% ee) and 20% of *cis*-(−)-**3a** (>95% ee).

With the advent of monochromatic light-emitting diodes, precision photochemistry has been advanced as an approach for providing greater control of photochemical reactions [37]. The significantly different product mixtures obtained from the 395 nm and 365 nm LED light sources demonstrate the importance of precision photochemistry for optimization of desired compounds. These results can be rationalized in terms of an initial oxadi-π-methane (OPDM) rearrangement to give diastereomeric bicyclic cyclopropanols **5** and **6** [47,48,49] (Scheme 2). We could not isolate the proposed cyclopropane intermediates. However, cyclopropanols are well known to undergo ring conversion to acyclic products [50]. Ring opening of the cyclopropanol results in isohumulones **2a** and **3a**. The OPDM cycloaddition between the sigma 3-4 bond and the p 5-6 bond can occur from either face of the cyclohexanone ring. Based on the product results, at 395 nm, the cycloaddition can be rationalized as stereospecific, resulting in bicyclic intermediate **5**. Ring opening of the cyclopropanol by proton transfer results in a single product (−)-(4*S*,5*S*)-**2a**. We propose that at the lower wavelength of 365 nm, a mixture of bicyclic intermediates **5** and **6** is obtained. A similar mixture of isomeric products has been reported for the photochemical OPDM rearrangement of 2,5-cyclohexadien-1-one and steroid intermediates [51,52]. The cyclopropanol ring opening of intermediate **6** is not stereospecific and results in a mixture of *trans* and *cis* isomers (+)-**2a** and (−)-**3a**, respectively.

### 2.4. Absolute Configuration of (−)-***2a*** by X-Ray Crystallography

**Figure 5:**
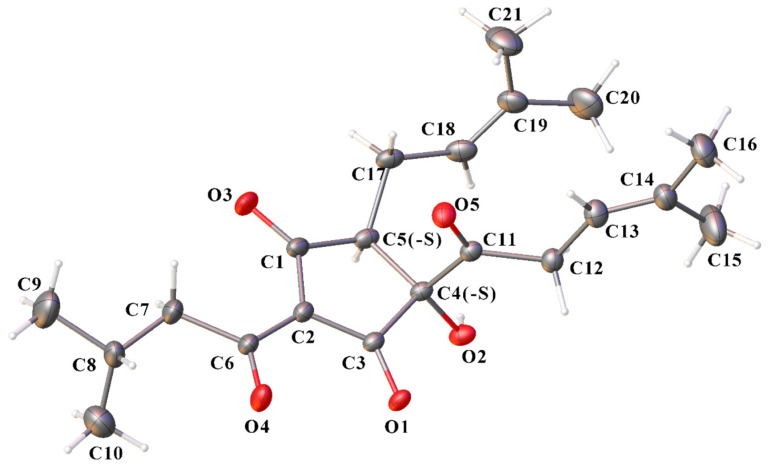
Molecular structure of *trans*-*n*-isohumulone showing displacement ellipsoids anion drawn at 50% probability level.

The absolute configuration of (−)-(*S*)-**1a** and the *cis* isomer (+)-(4*S*,5*R*)-**3a** have been previously established by X-ray crystallography of suitable salts with chiral amines [40]. Absolute configuration of the *trans*-isohumulones (−)-**2a** was inferred based on analysis of the hydrogenated derivative tetrahydro-n-isohumulone [40]. To further establish the absolute configuration of (−)-**2a**, we evaluated a set of salts prepared from seven chiral amines for potential direct X-ray analysis. Four of the salt pairs resulted in isolated solids; however, in all but one case, the solids were amorphous and not amenable to X-ray analysis. The crystallization of (−)-**2a** with (*S*)-2-benzylamino-1-phenylethanol afforded a highly crystalline salt suitable for X-Ray analysis (Figure 5). The hydroxyl hydrogen atom at the C3 position dissociated the *trans*-*n*-isohumulone and protonated at the amino N-atom of the 2-benzylamino-1-phenylethanol molecule. The absolute configuration of the humulone molecule is mainly determined by collecting the X-Ray intensity data using Cu-Kα radiation, which triggers the anomalous dispersion effect in the crystal. The *trans*-*n*-isohumulone:(*S*)-2-benzylamino-1-phenylethanol salt crystallizes in a non-centrosymmetric P2_1_2_1_2_1_ space group, with a refined Flack parameter of 0.14(9), which confirms the compound is enantiopure in nature. The molecular structure of *trans*-*n*-isohumulone anion is shown in Figure 5. It is observed that both the chiral ring carbon atoms C4 and C5 of the humulone molecule exhibit an *S*-configuration, as observed in the previously reported structures [40,53]. Similarly, the lone chiral carbon atom of (*S*)-2-benzylamino-1-phenylethanol labeled as C28 exhibits the expected *S*-configuration. The central five-membered ring of the isohumulone molecule exhibits a chair conformation with calculated puckering parameters of q2 = 0.174(4) Å and ϕ2 = 265.8(3)° [54,55]. The substituted moiety at C4 atom lies in the axial position form the five-membered Cramer and Pople plane with an angle of 19.9(3)°, the oxygen atom O3 at C1 and the substitution at C2 atom lies in the equatorial position from the five-membered plane with an angle of 83.7(3)°, whereas the substitution at C5 atom lies between the axial and equatorial (bisextional) position from the five-membered plane with an angle of 56.6(3)°, respectively. These deviation of substitution moieties from the five-membered Cramer and Pople plane shows that the molecule exhibits a *trans*-conformation.

## 3. Materials and Methods

### 3.1. General Methods

Commercial reagents of high purity were purchased (Millipore Sigma, St. Louis, MO, USA and Fisher Scientific, Hampton, NH, USA) and used without further purification.

### 3.2. Synthesis of Racemic-n-humulone (***1a***)

A solution of 241 mg (0.512 mmole) of n-humulone phenylenediamine salt in 25 mL of CH_2_Cl_2_ was treated with 3 N HCl to afford the free acid. The organic layer was washed with H_2_O, dried with Na_2_SO_4_ and concd in vacuo to afford 187 mg of the free acid as a semi-solid. Without further purification, the free acid was dissolved in 6 mL of isooctane, transferred to a pressure tube and flushed with Ar gas for 5 min. The sealed tube was heated to 130 °C using an oil bath for 12 h. The reaction mixture was allowed to cool and concd in vacuo to give a viscous reddish-yellow oil. Purification by preparative reverse-phase chromatography (60% MeOH/40% H_2_O/0.1% TFA) afforded 60 mg (36%) of a yellow oil. Analytical characterization data (Supplementary Material) are consistent with the reported values for the naturally occurring (−)-**1a**.

### 3.3. Synthesis of Racemic trans-n-Isohumulone (***2a***)

A solution of 77 mg (0.21 mmole) of racemic *n*-humulone in 15 mL ethanol was prepared. The resultant 0.014 M solution was introduced to the continuous flow photoreactor (395 nm, 42 W) at a flow rate of 1.0 mL/min. The dual flow syringe pump was programmed to follow the introduction of the 0.014 M stock solution with a flush of 125 mL reagent alcohol. Product collection was initiated after the first 50 mL of void volume. Following the void volume, 90 mL of eluant was collected and concd in vacuo. The crude semi-solid was dissolved in 2.5 mL ethanol, treated with 2.5 mL water and placed in a freezer overnight. The product was collected and washed with a minimum amount of ethanol-water to afford 32 mg (41.6%) of a crystalline white-yellow solid. Analytical characterization data (Supplementary Material) are consistent with the reported values for (−)-**2a**.

## 4. Conclusions

In summary, we have developed useful methods for the analysis of enantiomeric composition of humulones and isohumulones. Knowledge of the enantiomeric composition of isohumulones is important for evaluating the bioactivity and pharmacokinetics of these compounds. It is also relevant for understanding the taste profile of these compounds as bittering agents in food and drinks. Preparation of racemic *n*- and *co*-humulone was readily achieved at elevated temperatures in isooctane, exhibiting first-order kinetics for racemization and a half-life for (*S*)-(−)-**1a** of 116 m at 130 °C. Enantiomers of humulone **1a** were readily separated on five different CSPs, with Whelk-O1 showing the best resolution and overall performance. Isohumulone **2a** was not resolvable on the Whelko-O1 bonded phase, but the enantiomers were resolved on the carbohydrate IC-3 CSP. The carbohydrate-based IC-3 CSP was employed for resolution of enantiomers of **2** and **3**. Basic, magnesium-catalyzed isomerization of **1a** resulted in partial racemization of the *cis*-**3a** isomer, resulting in 88% ee. However, the *trans*-**2a** isomer was obtained in greater than 95% ee or enantiomerically pure within the limits of detection by chiral HPLC. Surprisingly, isomerization of **1a** in refluxing aqueous acetate buffer and pH of 5.5, a system chosen to mimic the pH and conditions of wort in beer brewing, resulted in both **2a** and **3a** in greater than 95% ee. The continuous photoflow isomerization of **1a** using a 395 nm LED light source resulted in exclusive formation of the *trans*-(−)-(4*S*,5*S*)-**2a** isomer in greater than 95% ee. X-ray crystallography of the salt obtained from (−)-**2a** and (*S*)-2-benzylamino-1-phenylethanol allowed confirmation of the absolute configuration as 4*S*,5*S*. Surprisingly, continuous photoflow isomerization using a 365 nm LED light source resulted in a mixture of three isomers: (−)-**2a**, (+)-**2a** and (−)-**3a**. This mixture consists of an 80:20 ratio of the *trans* and *cis* isomers, in which the *trans*-(−)-**2a** isomer is obtained in 46% ee and (−)-**3a** is obtained in 95% ee. The *cis*-(−)-**3a** isomer thus obtained has the opposite absolute configuration compared to thermal aqueous isomerization in either basic or buffered solution. This is the first report of a synthetic method for preparation of *cis*-(−) (4*R*,5*S*)-**3a**. The mechanistic implications for isomerization of *n*-humulone and other homologs have yet to be determined by more detailed computational methods. However, it is evident that the composition of the isomeric isohumulone products can be controlled by precision photochemistry [37]. These methods can be applied to understand the relevance of enantiomeric purity to biological evaluation of humulone and isohumulone-derived products.

## Figures and Tables

**Table 1:** HPLC separation factors (α) and resolution (R) of humulones **1** and isohumulones **2** and **3** on selected chiral stationary phases (CSP).

Compound	CSP	Mobile Phase * (% IPA)	Capacity Factor (k’)	Alpha (α)	Resolution (R)
**1a**	Whelk-O1	2	0.49 (+)	1.73	5.0
	IA3	5	0.92 (+)	1.30	2.5
	IB3	2	1.20 (+)	1.62	4.0
	IC3	2	0.37 (+)	2.85	2.6
	ADH	5	1.27 (+)	1.27	1.6
**1b**	Whelk-O1	1	1.55 (+)	1.11	1.6
	IC3	1	1.70 (+)	1.25	0.5
**2a**	Whelk-O1	1	2.0	NS	
	IC3	1	1.56 (+)	1.72	2.3
**2b**	IC3	1	1.60	1.55	3.0
**3a**	IC3	1	2.20 (−)	1.32	3.8
**3b**	IC3	1	2.10 (−)	1.24	2.1

* Mobile phase consists of hexane/isopropanol (IPA) with added 0.1% TFA. Capacity factor indicates the retention of the first eluted enantiomer, with its sign of rotation listed in parentheses. NS indicates no separation. Details for HPLC conditions and description of CSPs in Supplementary Material.

**Table 2:** Enantiomeric composition of isohumulones obtained from isomerization reactions under thermal or photochemical conditions.

Conditions	*trans*:*cis* Ratio (Isolated Yield) ^1^	*trans*-2a% ee (Rotation)	*cis*-3a% ee (Rotation)
5 eq MgSO_4_/2 eq NaOH (CH_2_Cl_2_/H_2_O), 10 °C, 5 d	17:83 (86%)	>95% (−)	88% (+)
5 eq MgSO_4_/2 eq NaOH (MeOH/H_2_O), heat, 15 m	43:57 (72%)	>95% (−)	88% (+)
Acetate buffer, pH = 5.5, reflux, 4 h	27:71 (80%) ^2^	>95% (−)	>95% (+)
Photoflow, 395 nm	97% *trans* (93%)	>95% (−)	3%
Photoflow, 365 nm	80:20 (53%)	46% (−)	>95% (−)

^1^ The isolated yield is listed in parentheses. All reactions were carried out until greater than 95% of conversion of the starting material was observed. ^2^ Yield determined by HPLC comparison to a standard curve. Reaction details given in the Supplementary Material and in previous references [35,36].

## Data Availability

Data are contained within the article and Supplementary Materials.

## Supplementary Materials

The following supporting information can be downloaded at: https://www.mdpi.com/article/10.3390/molecules31152731/s1. Figure S1: Photoreactor with 365 nm LEDs, computer-controlled syringe pumps, reactor cooling blocks, recirculation bath and Gilson fraction collector; Figure S2: Molecular structure of *trans*-*n*-isohumulone: (*S*)-2-benzylamino-2-phenylethanol salt (**4**) showing displacement ellipsoids anion drawn at 50% probability level; Table S1: Properties of amine salts with compound **2a**; Table S2: Crystal Structure refinement details of compound **4**. References [35,36,56,57,58,59,60] are cited in the supplementary materials.

## References

[1] Briggs D.E., Boulton C.A., Brookes P.A., Stevens R. (2004). Brewing Science and Practice.

[2] DeKeukeleire D. (2017). A happy, hoppy Odyssey: From a flavorsome hobby to a dream job. J. Am. Soc. Brew. Chem..

[3] Ting P.L., Ryder D.S. (2017). The bitter, twisted truth of the hop: 50 years of hop chemistry. J. Am. Soc. Brew. Chem..

[4] Almaguer C., Schonberger C., Gastl M., Arendt E., Becker T. (2014). *Humulus lupulus*—A story that begs to be told. J. Inst. Brew..

[5] Moir M. (2000). Hops—A millennium review. J. Am. Soc. Brew. Chem..

[6] Michiu D., Delvigne F., Mabon N., Jimbororean M., Fogarasi M., Mihai M., Tofana M., Thonart P. (2019). Inhibitory Effects of iso-α and β hop acids against *Pediococcus pentosaceus*. Not. Bot. Horti Agrobot..

[7] Steenackers B., De Cooman L., De Vos D. (2015). Chemical transformations of characteristic hop secondary metabolites in relation to beer properties and the brewing process: A review. Food Chem..

[8] Simpson W. (1993). Cambridge Prize Lecture. Studies on the sensitivity of lactic acid bacteria to hop bitter acids. J. Inst. Brew..

[9] Lafontaine S., Senn K., Dennenlohr J., Schubert C., Knoke L., Maxminer A.C., Rettberg N., Heymann H. (2020). Characterizing volatile and nonvolatile factors influencing flavor and American consumer preference toward nonalcoholic beer. ACS Omega.

[10] Buckett L., Schinko S., Urmann C., Riepl H., Rychlik M. (2020). Stable isotope dilution analysis of the major prenylated flavonoids found in beer, hop tea, and hops. Front. Nutr..

[11] Hagemann M.H., Bogner K., Marchioni E., Braun S. (2016). Chances for dry-hopped non-alcoholic beverages?. Brew. Sci..

[12] Lin M., Xiang D., Chen X., Huo H. (2019). Role of characteristic components of *Humulus lupulus* in promoting human health. J. Agric. Food Chem..

[13] Hurth Z., Faber M.-L., Fendrich F., Holzer M., Haarhaus B., Cawelius A., Schwabe K., Schempp C.M., Wolfle U. (2022). The anti-inflammatory effect of *Humulus lupulus* extract in vivo depends on the galenic system of the topical formulation. Pharmaceuticals.

[14] Koetter U., Biendl M. (2010). Hops (*Humulus lupulus*): A review of its historic and medicinal uses. J. Amer. Bot. Counc..

[15] Aghamiri V., Mirghafourvand M., Mohammad-Alizadeh-Charandabi S., Nazemiyeh H. (2016). The effect of hop (*Humulus lupulus L.)* on early menopausal symptoms and hot flashes: A randomized placebo-controlled trial. Complement. Ther. Clin. Pract..

[16] Walker E., Lo K., Pahl M., Shin H., Lang C., Wohlers M., Poppitt S., Sutton K., Ingram J. (2022). An extract of hops (*Humulus lupulus* L.) modulates gut peptide hormone secretion and reduces energy intake in healthy-weight men: A randomized, crossover clinical trial. Am. J. Clin. Nutr..

[17] Sun S., Wang X., Yuan A., Liu J., Li Z., Xie D., Zhan H., Luo W., Xu H., Liu J. (2021). Chemical constituents and bioactivities of hops (*Humulus lupulus* L.) and their effects on beer-related microorganisms. Food Energy Secur..

[18] Karabin M., Hudcova T., Jelinek L., Dostalek P. (2016). Biologically active compounds from hops and prospects for their use. Compr. Rev. Food Sci. Food Saf..

[19] Van Cleemput M., Cattoor K., De Bosscher K., Haegeman G., De Kekeuleire D., Heyerick A. (2009). Hop (*Humulus lupulus)*-derived bitter acids as multipotent bioactive compounds. J. Nat. Prod..

[20] Hieronymus S. (2012). For the Love of Hops.

[21] Benkherouf A., Eerola K., Soini S., Uusi-Oukari M. (2020). Humulone modulation of GABA_A_ receptors and its role in hops sleep-promoting activity. Front. Neurosci..

[22] Tyrrell E., Archer R., Skinner G., Singh K., Colston K., Driver C. (2010). Structure elucidation and an investigation into the in vitro effect of hop acids on human cancer cells. Phytochem. Lett..

[23] Verzele M., DeKeukeleire D. (1991). Chemistry and Analysis of Hop and Beer Bitter Acids.

[24] Ponticelli M., Russo D., Faraone I., Sinisgalli C., Labanca F., Lela L., Milelli L. (2021). The promising ability of *Humulus lupulus* L. iso-α-acids vs. Diabetes, Inflammation, and Metabolic Syndrome: A systematic review. Molecules.

[25] Obara K., Mizutani M., Hitomi Y., Yajima H., Kondo K. (2009). Isohumulones, the bitter component of beer, improve hyperglycemia and decrease body fat in Japanese subjects with prediabetes. Clin. Nutr..

[26] Ting P., Pratt J., Ryder D. (2011). Resolution of (+/−)-Tetrahydrocohumulones and Properties of Their Isomerized Derivatives. J. Am. Soc. Brew. Chem..

[27] Vanhoenacker G., De Keukeleire D., Sandra P. (2004). Analysis of *iso*-α-acids and reduced *iso*-α-acids in beer by direct injection and liquid chromatography with ultraviolet absorbance detection or with mass spectroscopy. J. Chromatogr. A.

[28] Desai A., Konda V., Darland G., Austin M., Prabhu K., Bland J., Carroll B., Tripp M. (2009). META060 inhibits multiple kinases in the NF-kB pathway and suppresses LPS—Mediated inflammation in vitro and ex vivo. Inflamm. Res..

[29] Finlin B., Zhu B., Kok B., Godia C., Westgate P., Grayson N., Sims R., Bland J., Saez E., Kern P. (2017). The influence of a KDT501, a novel isohumulone, on adipocyte function in humans. Front. Endocrinol..

[30] Bland J., Grayson N., Wolfe A., Wu S. (2018). Use of Isohumulones and Derivatives Thereof to Treat Polycystic Ovary Syndrome. WO.

[31] Bland J.S., Minich D., Lerman R., Darland G., Lamb J., Tripp M., Grayson N. (2015). Isohumulones from hops (*Humulus lupulus*) and their potential role in medical nutrition therapy. Pharma Nutr..

[32] Hamper B.C., Meisel J.W. (2020). Introducing nonscience majors to science literacy via a laboratory and lecture beer brewing course. J. Chem. Ed..

[33] Hamper B., Zawatzky K., Zhang V., Welch C.J. (2017). Rapid determination of humulones and isohumulones in beers using MISER LC-MS analysis. J. Am. Soc. Brew. Chem..

[34] Hamper B., Viriyasiri N., Boland A., Espinosa L., Campbell H., McKeever M. (2021). Liquid chromatography-mass spectrometry (LC-MS) analysis of hop-derived humulone and isohumulone constituents in beer: The bitter truth of hops utilization during brewing. LC-GC Mag. N. Am..

[35] Hamper B., Campbell H., Luo R., Murphy M., Gleason P., Smith T., Jagan R. (2024). Selective synthesis of deuterated cis- and trans-isohumulones and trans-isohumulinones. Synthesis.

[36] Hamper B.C., Gallow B., Giovine G., Smith T. (2025). Continuous-flow photochemical isomerization of humulones to isohumulones. Molecules.

[37] Pashley-Johnson F., Wu X., Carroll J., Walden S., Frish H., Unterreiner A.-N., Du Prez F., Wagenknecht H.-A., Alaniz J., Feringa B.L. (2025). Precision Photochemistry: Every Photon Counts. Angew. Chem. Int. Ed..

[38] Clarke B., Hildebrand M. (1965). The isomerization of humulone: I. Isolation of photoisohumulone. J. Inst. Brew..

[39] Sharpe F., Ormrod I. (1991). Fast isomerization of humulone by photoreaction: Preparation of an HPLC standard. J. Inst. Brew..

[40] Urban J., Dahlberg C., Carroll B., Kaminski W. (2013). Absolute configuration of Beer’s bitter compounds. Angew. Chem. Int. Ed..

[41] Verzele M., Van Boven M. (1971). The isomerization mechanism of humulone. Bull. Soc. Chim. Belg..

[42] Anteunis M., Verzele M. (1959). Racemic Tetrahydrohumulone. Bull. Soc. Chim. Belg..

[43] Pirkle W., Welch C., Lamm B. (1992). Design, synthesis and evaluation of an improved enantioselective naproxen selector. J. Org. Chem..

[44] Simpson W. (1993). Ionization behaviour of hop compounds and hop-derived compounds. J. Inst. Brew..

[45] Koller H. (1969). Magnesium ion catalyzed isomerization of humulone: A new route to pure isohumulone. J. Inst. Brew..

[46] Malowicki M.G., Shellhammer T.H. (2005). Isomerization and degradation kinetics of hop (*Humulus lupulus*) acids in a model wort-boiling system. J. Agric. Food Chem..

[47] Dong Q.-X., Ke Y.-H., Zhang Y., Huang H.-M. (2025). Photochemical di-π-methane rearrangement reactions. Ang. Chem. Int. Ed..

[48] Demuth M., Padwa A. (1991). Synthetic Aspects of the Oxadi-π-Methane Rearrangement. Organic Photochemistry.

[49] De Keukeleire D., Blondeel G.M. (1979). The mechanism of the regio- and stereospecific photorearrangement of humulone to the beer bitter component *trans* isohumulone. Tetrahedron Lett..

[50] Laktsevich-Iskryk M., Hurski A., Oseka M., Kananovich D. (2025). Recent advances in asymmetric synthesis via cyclopropanol intermediates. Org. Biomol. Chem..

[51] Schultz A.G., Lavieri F.P., Macielag M., Plummer M. (1987). 2,5-Cyclohexadien-1-one to bicyclo[3.1.0]hexenone photorearrangement. Development of the reaction for use in organic synthesis. J. Am. Chem. Soc..

[52] Zimmerman H.E., Amesto D. (1996). Synthetic aspects of the di-p-methane rearrangement. Chem. Rev..

[53] Intelmann D., Kummerlöwe G., Haseleu G., Desmer N., Schulze K., Fröhlich R., Frank O., Luy B., Hofmann T. (2009). Structures of Storage-Induced Transformation Products of the Beer’s Bitter Principles, Revealed by Sophisticated NMR Spectroscopic and LC–MS Techniques. Chem.—A Eur. J..

[54] Nauwelaerts K., Lescrinier E., Sclep G., Herdewijn P. (2005). Cyclohexenyl nucleic acids: Conformationally flexible oligonucleotides. Nucleic Acids Res..

[55] Chan L., Hutchison G., Morris G. (2021). Understanding Ring Puckering in Small Molecules and Cyclic Peptides. J. Chem. Inf. Model..

[56] Dong M.W. (2006). Modern HPLC for Practicing Scientists.

[57] Bruker (2016). APEX3, SAINT-Plus and SADABS (2016).

[58] Sheldrick G.M. (2015). Crystal structure refinement with SHELXL. Acta Crystallogr. C.

[59] Spek A.L. (2009). Structure validation in chemical crystallography. Acta Crystallogr. Sect. D.

[60] Dolomanov O.V., Bourhis L.J., Gildea R.J., Howard J.A.K., Puschmann H. (2009). OLEX2: A complete structure solution, refinement and analysis program. J. Appl. Crystallogr..

