# Supplementary material for: Enantioselectivity of Isomerization of n-Humulone to trans- and cis-n-Isohumulones
Source: Molecules. 2026 Aug 6;31(15):2731. doi: 10.3390/molecules31152731 (PMC13467588; doi:10.3390/molecules31152731)
Supplement: Supplementary file 1 [file molecules-31-02731-s001.zip › molecules-4432203-supplementary.pdf]

Enantioselectivity of Isomerization of *n*-Humulone to *trans*- and *cis*-*n*-isohumulone

Bruce C. Hamper, Gregory Giovine, Rajamoni Jagan and Trevor Smith*

University of Missouri-St. Louis, Department of Chemistry & Biochemistry, 43 Benton Court, St. Louis, MO 63122, USA

hamperb@umsl.edu

Contents

1. Experimental Methods

Methods and Materials 3

2. Synthetic Methods

(+/-)-*n*-humulone (**1a**) 5
(+/-)-trans *n*-isohumulone (**2a**) 7
(+/-)-cis *n*-isohumulone (**3a**) 9
(+/-)-*co*-humulone (**1b**) 11
(+/-)-trans *co*-isohumulone (**2b**) 12
(+/-)-cis *co*-isohumulone (**3b**) 14
(-)-(4S, 5S)-trans-n-isohumulone and (S)-2-(benzylamino)-
1-phenylethanol salt (1:1) (**4**) 15

3. Crystallographic Data (X-RAY) 16

4. References 19

5. NMR Data 20-28

1. **Experimental Methods**

Materials and Methods

Commercial reagents of high purity were purchased (Millipore Sigma, USA and Fisher Scientific, Hampton, NH) and used without further purification. Supercritical CO_2 hops extract (Batch AU-401) was obtained from Hopsteiner (S.S.Steiner, Yakima, Washington, USA). Deionized water used for chromatography was purified by means of a Milli-Q Gradient A10 system (Millipore, USA).IR spectra were obtained by FTIR-ATR and reported in reciprocal wavenumbers (cm^{-1}). ^1H NMR spectra were referenced to residual $CHCl_3$ (7.27 ppm) or C_6D_6 (7.16 ppm). ^{13}C NMR spectra were referenced to the center line of $CDCl_3$ (77.2 ppm) or C_6D_6 (128.4 ppm). Reverse phase HPLC analysis was conducted with an Agilent 1200 system equipped with UV detection. Mobile phase solvents were prepared from HPLC grade 0.1% TFA in H_2O (Mobile Phase A) and 0.1% TFA in CH_3CN (Mobile Phase B). A Poroshell 120 EC-C18 column (3.0 mm id x 150 mm; 2.7 um particle size) was employed using a flow rate of 0.4 mL/min and a gradient of 50%A/50% B from 0 – 2 min, 50% B to 100% B from 2 – 12 min followed by a hold of 100% B for 8 minutes. Preparative reverse phase chromatography was carried out using a C18 column (10 um particle diameter; 250 mm x 20 mm i.d.) and isocratic mobile phases consisting of mixtures of HPLC grade methanol and H_2O at a flow rate of 10 mL/min.

Analytical normal phase chiral HPLC was performed using a Gilson 307 pump equipped with a 5 mL pump head, Rheodyne 7125 injector with 20 uL injection loop and a sequential Knauer UV detector followed by an ALP Advanced Laser Polarimeter which provided rotational data at 635 nm (ALP, PDR-Chiral, Inc.). 'No Signal' Indicates that the rotation of the analyte was below the detectable limit for the polarimetric detector. Data collection was achieved using Peak Simple SRI Model 302 data collection system (SRI Instruments, CA, USA). HPLC columns with 5 um particle size chiral stationary phases (CSP) were 250 mm x 4.6 mm I.D and contained either Chiralpak AD-H (Chiral Technologies, West Chester, PA, USA) or Pirkle Covalent (R,R) Whelk-O 1 (Regis Chemical Co., Morton Grove, IL, USA). The HPLC columns with 3 um size CSP were 100 x 4.6 mm I.D. and contained ChiralPak either IA-3, IB-3, IC-3 or AS-3. Chromatographic runs were performed at a flow rate of 2.0 mL/min for the longer 250 mm columns and 1.0 mL/min for the shorter 100 mm columns. Column temperature was controlled at 20 ^oC using a digital Peltier temperature controller (Cera 250, Cobert Associates, USA). Mobile phase was prepared using mixtures of hexanes and isopropyl alcohol containing 0.1 % trifluoroacetic acid. Capacity factors (k') indicate the retention of the first eluting enantiomer. Separation factors (a) were calculated as a ratio of capacity factors for the two eluting enantiomers. Resolution was calculated from the base peak widths of the eluted peaks, where $t_{R2} – t_{R1}$ indicates the difference in retention time of the two peaks and W is the width of the corresponding peaks [1].

$$R_s = \frac{t_{R2} - t_{R1}}{\left(\frac{W_{b2} + W_{b1}}{2}\right)}$$

Continuous flow photoreactors were constructed using a cylindrical base, strip LEDs and UV transparent THV tubing (0.25 in od x 5/32" id, www.mcmaster.com, #5737K41) as previously described [2] The two units used for this study used either 395 nm or 365 nm Waveform LEDs (waveform.com; Part # 7021.95.5M and #7021.65.5M, respectively). The 395 nm reactor consisted of 348 LEDs having a total wattage of 41.8 W, 442 cm of THV tubing and a void volume of 57 mL. The Waveform 365 unit consists

of 351 LEDs, total wattage of 42.1 W and 510 cm of THV tubing for a void volume of 66 mL. Cooling was provided by a set of liquid cooling copper pipes imbedded in an aluminum block and connected to a glycol-water recirculation chiller bath. The chiller was set to a temperature of 10 °C which provided a constant photoreactor temperature of 23-24 °C.

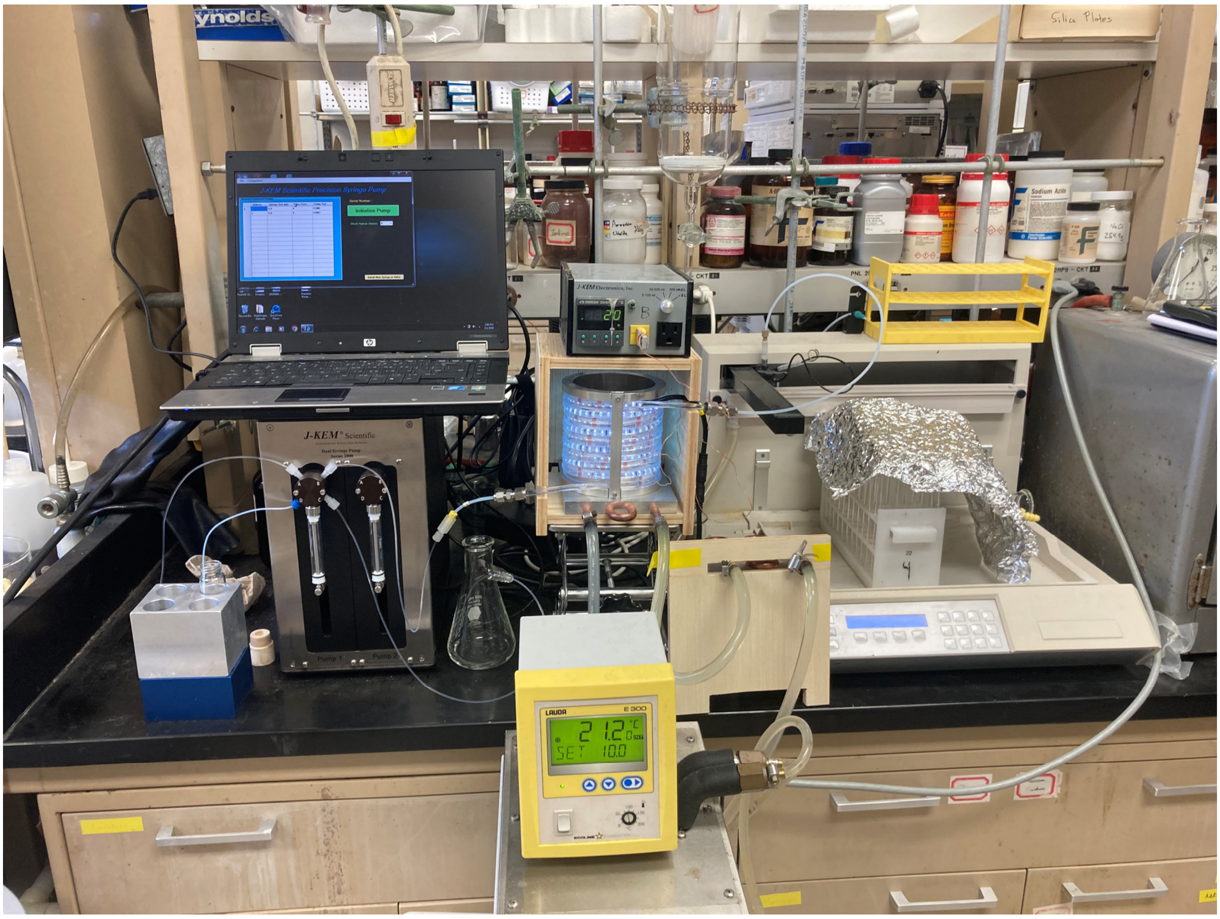

Figure S1. Photoreactor with 365 nm LEDs, computer-controlled syringe pumps, reactor cooling blocks, recirculation bath and Gilson fraction collector.

2. Synthetic Methods

(+/-)-n-Humulone (1a)

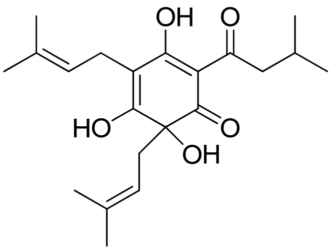

A solution of 241 mg (0.512 mmole) of n-humulone phenylenediamine salt in 25 mL of CH_2Cl_2 was treated with 3N HCl to afford the free acid. The organic layer was washed with H_2O, dried with Na_2SO_4 and concd in vacuo to afford 187 mg of the free acid as a semi-solid. Without further purification, the free acid was dissolved in 6 mL of isooctane, transferred to a pressure tube and flushed with Ar gas for 5 min. The sealed tube was heated to 130 °C using an oil bath for 12 hrs. The reaction mixture was allowed to cool and concd in vacuo to give a viscous reddish-yellow oil. Purification by preparative reverse phase chromatography (60% MeOH/40% H2O/0.1% TFA) afforded 60 mg (36%) of a yellow oil.

HPLC (UV 230 nm): 13.29 min (98%).

UV (0.1%TFA in 80% CH_3CN/H_2O): λ_{max} = 234, 322 nm;

^{1}H NMR ($CDCl_3$, 300 MHz): δ 7.28 (brs, 1H), 5.13 (t, 1H, *J* = 7.0 Hz), 4.99 (t, 1H, *J* = 7.6 Hz), 4.38 (brs, 1H), 3.04-3.10 (m, 2H), 2.77 (d, 2H, *J* = 6.8 Hz), 2.45-2.55 (m, 2H), 2.14 (septet, 1H, *J* = 6.5 Hz), 1.73 (s, 3H), 1.68 (s, 6H), 1.52 (s, 3H), 1.00/0.97 (d, 3H each, *J* = 6.5 Hz).

^{13}C NMR ($CDCl_3$, 75 MHz): δ 200.3, 195.3, 190.9, 167.8, 138.2, 132.8, 121.0, 115.8, 109.3, 106.0, 78.9, 46.5, 42.8, 26.4, 26.0, 25.8, 22.8, 22.6, 21.1, 17.9, 17.8.

MS (ESI-): *m/z* (%) 361 (M-1, 100).

HRMS (ESI-): *m/z* calcd for $C_{21}H_{29}O_5$ $(M-1)^{-1}$: 361.2022; found: 361.2014 (-2.3 ppm).

Chromatogram of (S)-(-)-n-humulone. CSP Regis (R,R) WhelkO 1; 1% IPA/hexanes/0.1% TFA.

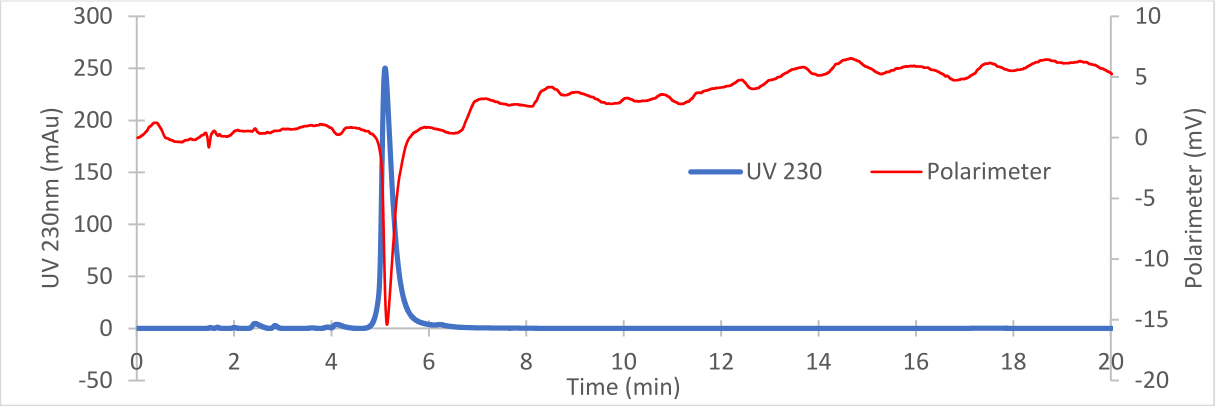

	Retention Time (min)	Area	% Area	Rotation
1	4.106	62.6	1.4	No signal
2	5.10	4354.1	98.6	-

No Signal observed for the minor 4.10 min peak; below limits of detection.

Chromatogram of racemic n-humulone; CSP Regis (R,R) WhelkO 1; 1% IPA/hexanes/0.1% TFA

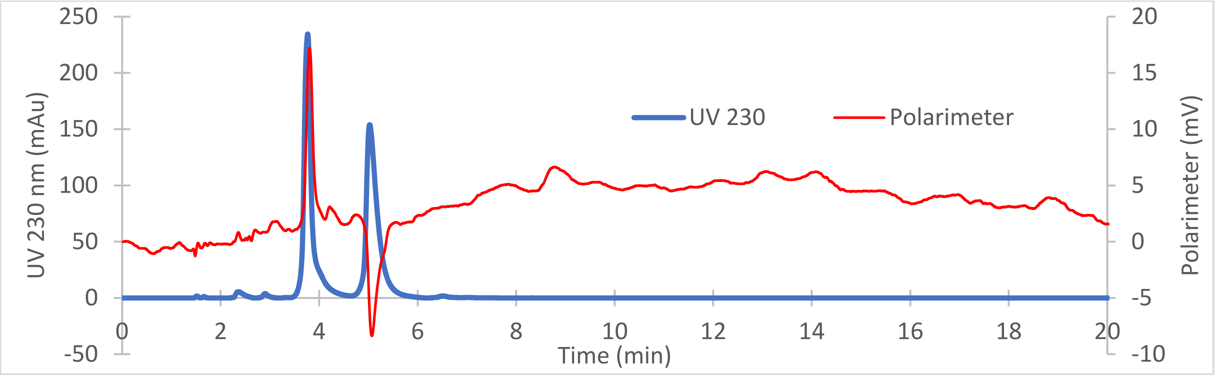

	Retention Time (min)	Area	% Area	Rotation
1	3.766	2668.9	50.9	+
2	5.026	2572.8	49.1	-

(+/-)-trans *n*-isohumulone (2a)

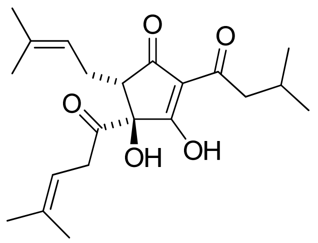

A solution of 77 mg (0.21 mmole) of racemic *n*-humulone in 15 mL ethanol was prepared. The resultant 0.014 M solution was introduced to the continuous flow photoreactor (395 nm, 42 W) at a flow rate of 1.0 mL/min. The dual flow syringe pump was programmed to follow the introduction of the 0.014 M stock solution with a flush of 125 mL reagent alcohol. Collection of product was initiated after the first 50 mL of void volume. Following the void volume, 90 mL of eluant was collected and concd in vacuo. The crude semi-solid was dissolved in 2.5 mL ethanol, treated with 2.5 mL water and placed in a freezer overnight. The product was collected and washed with a minimum amount of ethanol-water to afford 32 mg (41.6%) of a crystalline white-yellow solid.

mp 68-69 °C.

HPLC (UV 230 nm): 12.17 min (98%).

UV (0.1%TFA in 80% CH_3CN/H_2O): λ_{max} = 226, 282 nm;

FTIR (ATR): 3407 (m, br), 2925 (m), 1697 (s), 1626 (s), 1548 (s).

^1H NMR (C_6D_6, 300 MHz): δ 0.75 (d, 3H, *J* = 6.7 Hz), 0.79 (d, 3H, *J* = 6.7 Hz), 1.43 (s, 3H), 1.44 (s, 3H), 1.58 (s, 6H), 2.01 (septet, 1H, *J* = 6.7 Hz), 2.50-2.70 (m 4H), 2.81 (dd, 1H, *J* = 5.7 Hz, 10.1 Hz), 3.38-3.41 (m, 2H), 5.23 (t, 1H, *J* = 5.6 Hz), 5.45 (t, 1H, *J* = 6.1 Hz).

^{13}C NMR (C_6D_6, 75 MHz): δ 18.2, 18.4, 22.6, 22.8, 24.2, 26.0, 26.1, 26.9, 39.4, 44.7, 56.0, 91.4, 111.1, 115.9, 121.5, 134.6, 135.9, 196.4, 198.1, 205.7, 207.3.

MS (ESI-): *m/z* (%) 361 (M-1, 100), 377 (95)

HRMS (ESI-): *m/z* calcd for $C_{21}H_{29}O_5$ $(M-1)^{-1}$: 361.2022; found: 361.2026 (+1.1 ppm).

Anal. Calcd for $C_{21}H_{30}O_5$: C, 69.59; H, 8.34. Found: C, 69.55; H, 8.41.

Chromatogram of (4S,5S)-(-)-trans-n-isohumulone; CSP IC-3; 1% IPA/hexanes/0.1% TFA

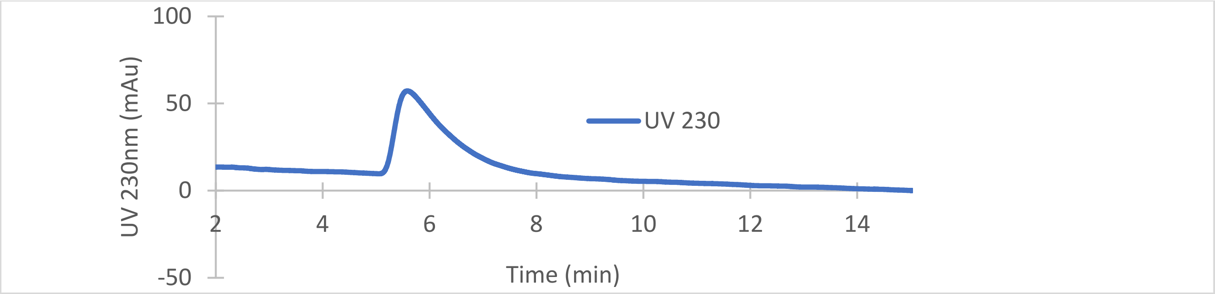

	Retention Time (min)	Area	% Area	Rotation
1	5.586	3548.9	>95%	No signal

No Signal – Rotation of (-)-2a was not observed by the chromatographic polarimetric detector

Chromatogram of racemic trans- n-isohumulone; CSP IC-3; 1% IPA/hexanes/0.1% TFA

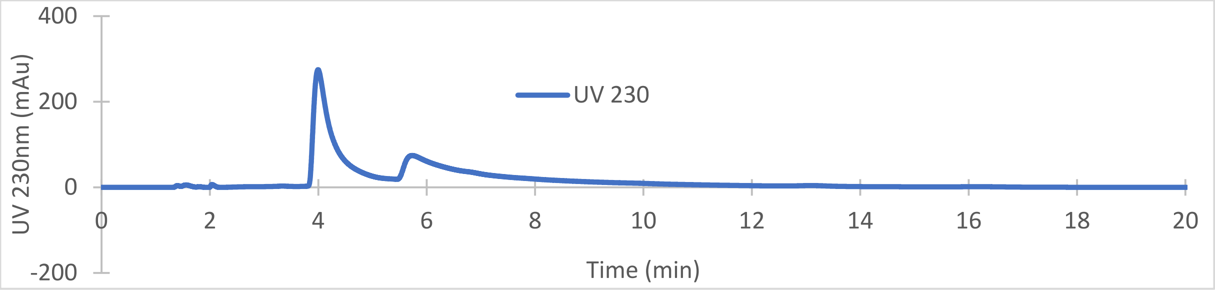

	Retention Time (min)	Area	% Area	Rotation
1	3.993	7632.9	47.4	No signal
2	5.733	8471.0	52.6	No signal

(+/-)-cis *n*-isohumulone (3a)

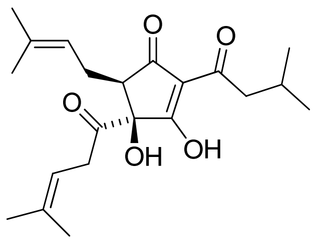

A solution of 81 mg (0.22 mmole) of racemic *n*-humulone in 20 mL CH_2Cl_2 was cooled in an ice water bath and treated with 2.5 mL of 0.40 M NaOH. The mixture was subsequently treated dropwise with a solution of 135 mg $MgSO_4$ in 2 mL of H_2O and allowed to stir for 48 hrs. The volume of the reaction mixture was reduced by concd in vacuo to afford an aqueous slurry. The mixture was placed in the freezer overnight to induce solid precipitation. The resultant magnesium salt was collected by filtration and dried to afford 34.8 mg (39%; based on mw of Mg salt) of a tan solid. Analytical data was consistent with the previously reported (-)-cis-n-isohumulone [3]

mp 220 ^{o}C (Mg salt, dec)

HPLC (UV 280 nm): 12.87 min (91%); 12.63 min (trans isomer, 9%).

UV (0.1%TFA in 80% CH_3CN/H_2O): major peak 12.87 min;λ_{max} = 228, 276 nm;

^{1}H NMR ($CDCl_3$, 300 MHz): δ 9.27 (brs, 1H), 5.19 (t, 1H, *J* = 5.7 Hz), 5.00 (t, 1H, *J* = 7.3 Hz), 4.19 (brs, 1H), 3.30 (d, 2H, *J* = 6.7 Hz), 3.19 (t, 1H, *J* = 6.5 Hz), 2.78 (dd, 1H, *J* = 14.2, 6.9 Hz), 2.67 (dd, 1H, *J* = 14.3, 7.1 Hz), 2.50 (dd, 1H, *J* = 14.5, 7.3 Hz), 2.40 (dd, 1H, *J* = 14.5, 6.5 Hz), 2.12-2.16 (m, 1H), 1.72 (s, 3H), 1.63 (s, 3H), 1.59 (s, 3H), 1.57 (s, 3H), 0.93-1.02 (m, 6H).

MS (ESI-): *m/z* (%) 361 (M-1, 65), 347 (100).

HRMS (ESI-): *m/z* calcd for $C_{21}H_{29}O_5$ $(M-1)^{-1}$: 361.2022; found: 361.2030 (+2.2 ppm).

Chromatogram of (4S,5R)-(+)-cis-n-isohumulone; CSP IC-3; 1% IPA/hexanes/0.1% TFA

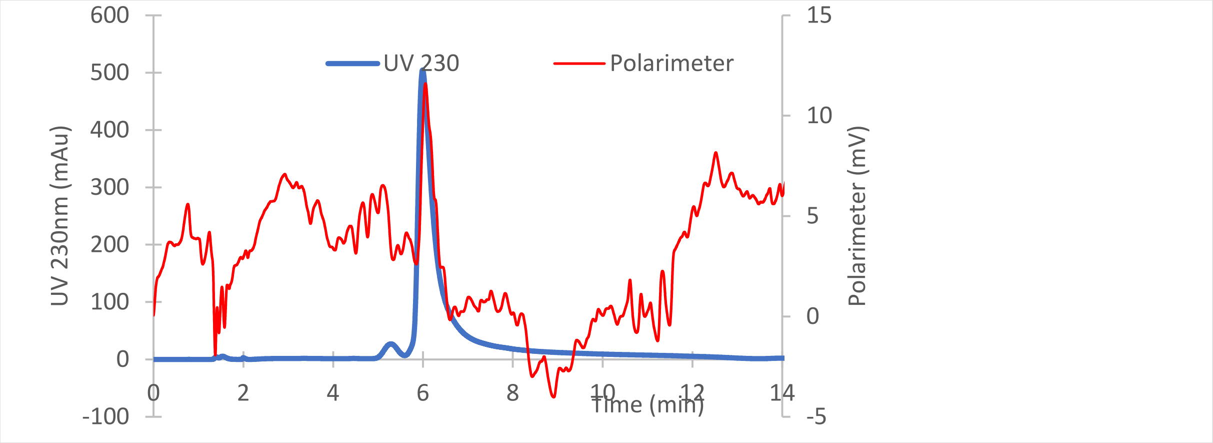

	Retention Time (min)	Area	% Area	Rotation
1	5.286	603.3	4	No signal
2	5.990	14591.1	96	+

Chromatogram of racemic cis- n-isohumulone; CSP IC-3; 1% IPA/hexanes/0.1% TFA

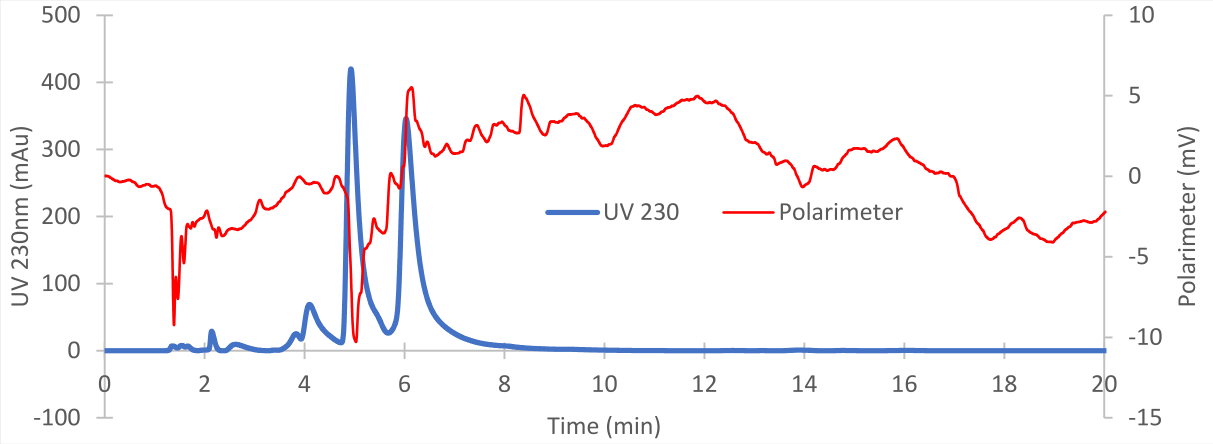

	Retention Time (min)	Area	% Area	Rotation
1	4.930	7412.1	46	-
2	6.030	8706.2	54	+

(+/-)-co-Humulone (1b)

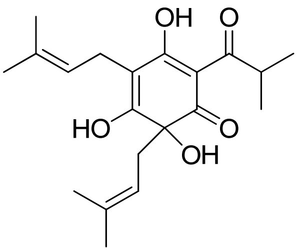

Racemization of (-)-cohumulone (-)-**1b** was carried out as described for n-humulone. A solution of 232 mg (0.66 mmol) of (-)-**1b** in 6 mL isooctane in a sealed tube was racemized by heating and purified by reverse phase chromatography to afford 90 mg (39%) of racemic cohumulone as a yellow oil. Analytical data was consistent with the previously reported (-)-cohumulone [3].

HPLC (UV 231 nm):12.61 min (91%)

UV (0.1%TFA in 80% CH_3CN/H_2O): λ_{max} = 234, 282, 324 nm;

^{1}H NMR ($CDCl_3$, 300 MHz): δ5.14 (t, 1H, *J* = 6.7 Hz), 5.03 (t, 1H, *J* = 7.6 Hz),4.83 (brs, 1H, OH), 3.70 (sep, 1H, *J* = 6.7 Hz), 3.12 (dd, 1H, *J* = 14.4, 7.3 Hz),3.03 (dd, 1H, *J* = 14.3, 7.2 Hz),2.60 (dd, 1H, *J* = 13.9, 7.8 Hz),2.50 (dd, 1H, *J* = 13.8, 7.9 Hz),1.73 (s, 3H),1.68 (s, 6H),1.52 (s, 3H),1.20 (d, 3H, *J* = 6.7 Hz),1.13 (d, 3H, *J* = 6.7 Hz).

MS (ESI-): *m/z* (%) 347 (M-1, 100)

HRMS (ESI-): *m/z* calcd for $C_{20}H_{27}O_5$ $(M-1)^{-1}$: 347.1866; found: 347.1871 (+1.6 ppm).

Chromatogram of (S)-(-)-co-humulone (file -188); CSP IC-3; 2% IPA/hexanes/0.1% TFA

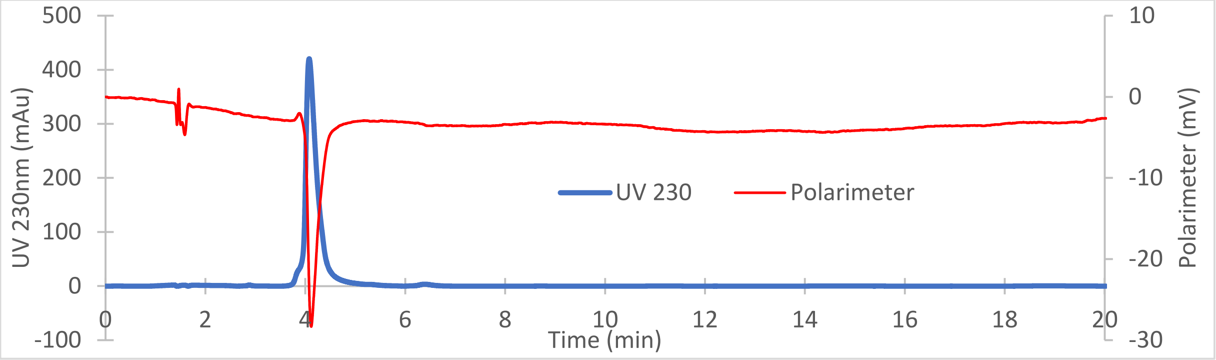

	Retention Time (min)	Area	% Area	Rotation
1	4.078	7132.3	>98%	-

Chromatogram of racemic co-humulone(file -162); CSP Regis (R,R) WhelkO 1; 1% IPA/hexanes/0.1% TFA

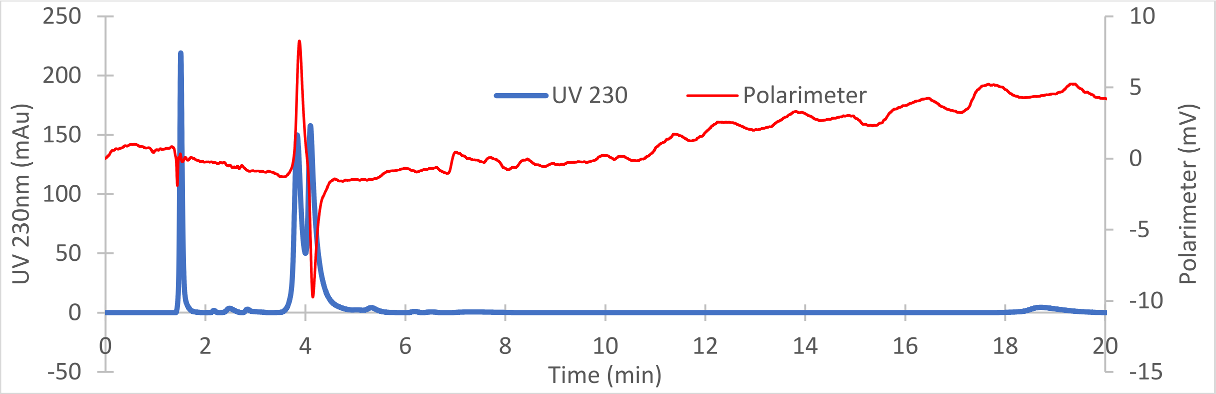

	Retention Time (min)	Area	% Area	Rotation
1	3.836	1573.0	44	+
2	4.100	2002.6	56	-

(+/-)-trans *co*-isohumulone (2b)

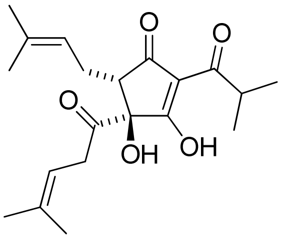

Continuous flow photochemical isomerization was carried out as previously described for (+/-)-trans-*n*-isohumulone. From 95 mg of racemic co-humulone was obtained 62 mg (81%) of a light yellow oil. Analytical data was consistent with the previously reported (-)-trans-*co*-humulone [3].

HPLC (UV 231 nm): 11.32 min (95%)

UV (0.1%TFA in 80% CH_3CN/H_2O): λ_{max} = 228, 273 nm;

^{1}H NMR ($CDCl_3$, 300 MHz): δ 5.19 (t, 1H, *J* = 6.4 Hz), 5.12 (t, 1H, *J* = 5.9 Hz), 4.10 (brs, 1H), 3.45-3.55 (m, 1H), 3.29 (d, 2H, *J* = 5.9 Hz), 3.04 (dd, 1H, *J* = 9.2, 6.3 Hz), 2.50-2.60 (m, 1H), 2.20-2.30 (m, 1H), 1.72 (s, 3H), 1.68 (s, 3H), 1.56 (s, 3H), 1.52 (s, 3H), 1.17 (d, 3H, *J* = 7.0 Hz), 1.13 (d, 3H, *J* = 7.0 Hz).

MS (ESI-): *m/z* (%) 347 (M-1, 100), 348 (50), 349 (10)

HRMS (ESI-): *m/z* calcd for $C_{20}H_{28}O_5$ $(M-1)^{-1}$: 347.1866; found: 347.1849 (-4.8 ppm).

Chromatogram of (4S,5S)-(-)-trans-co-isohumulone (file -188); CSP IC-3; 1% IPA/hexanes/0.1% TFA

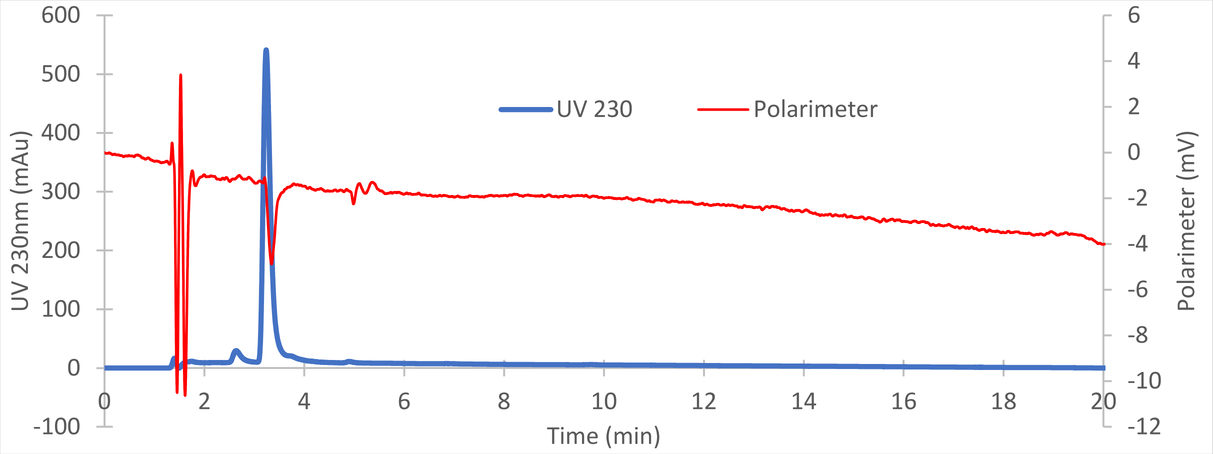

	Retention Time (min)	Area	% Area	Rotation
1	2.633	610	8%	No signal
2	3.236	6752	92%	-

Chromatogram of racemic trans- co-isohumulone (file -187); CSP IC-3; 2% IPA/hexanes/0.1% TFA

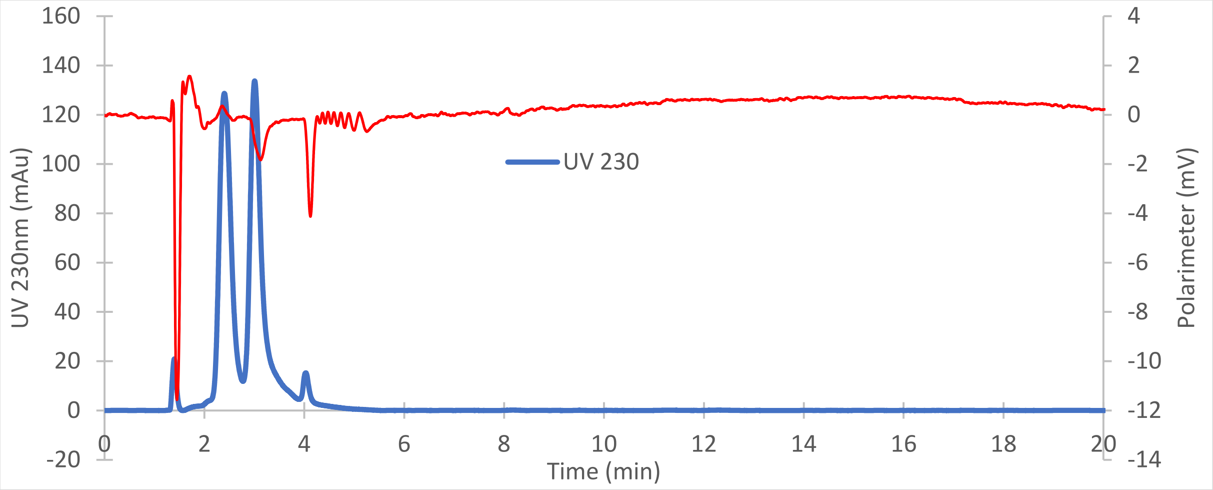

	Retention Time (min)	Area	% Area	Rotation
1	2.400	2262	48%	+
2	3.006	2466	52%	-

(+/-)-cis *co*-isohumulone (3b)

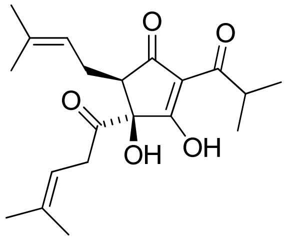

Compound was prepared following the method described for cis-*n*-isohumulone. From 56 mg of racemic *co*-humulone was obtained 30 mg (52%) of the magnesium salt of (+/-) cis-co-isohumulone as an off-white solid. Sample consisted of a mixture of cis and trans isomers (85:15). Analytical data was consistent with the previously reported (-)-cis-*co*-humulone [3].

mp 220 °C (Mg salt, dec)

HPLC (UV 280 nm) 11.56 min (85%, cis isomer), 11.35 min (15%, trans isomer).

UV (0.1%TFA in 80% CH_3CN/H_2O):λ_{max} = 232, 269.

^{1}H NMR ($CDCl_3$, 300 MHz): δ 5.20 (t, 1H, *J* = 6.3 Hz), 5.02 (t, 1H, *J* = 7.3 Hz), 3.46 (sep, 1H, *J* = 6.8 Hz), 3.32 (d, 2H, *J* = 6.7 Hz), 3.22 (t, 1H, *J* = 6.4 Hz), 2.48 (dd, 1H, *J* = 14.5, 7.2 Hz), 2.41 (dd, 1H, *J* = 14.4, 6.4 Hz), 1.72 (s, 3H), 1.64 (s, 3H), 1.59 (s, 3H), 1.57 (s, 3H), 1.11-1.16 (m, 6H).

MS (ESI-):*m/z*(%) 347 (M-1, 100), 348 (18), 349 (2).

HRMS (ESI-): *m/z* calcd for $C_{20}H_{28}O_5$ $(M-1)^{-1}$: 347.1866; found: 347.1855 (-3.1 ppm).

Chromatogram of (4S,5R)-(+)-cis-co-isohumulone (file -179); CSP IC-3; 1% IPA/hexanes/0.1% TFA

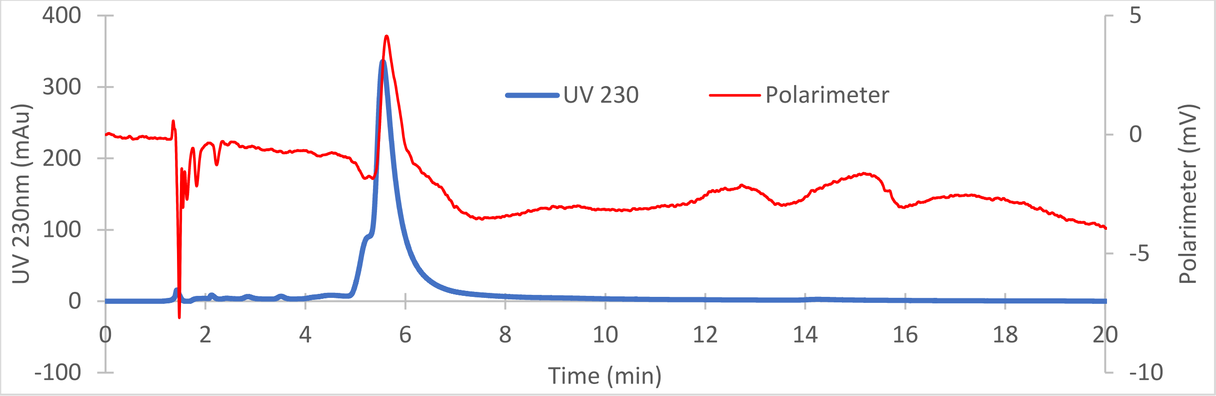

	Retention Time (min)	Area	% Area	Rotation
1	4.52	390	3%	No Signal
2	5.55	13239	97%	+

Chromatogram of racemic cis- co-isohumulone(file -180); CSP IC-3; 1% IPA/hexanes/0.1% TFA

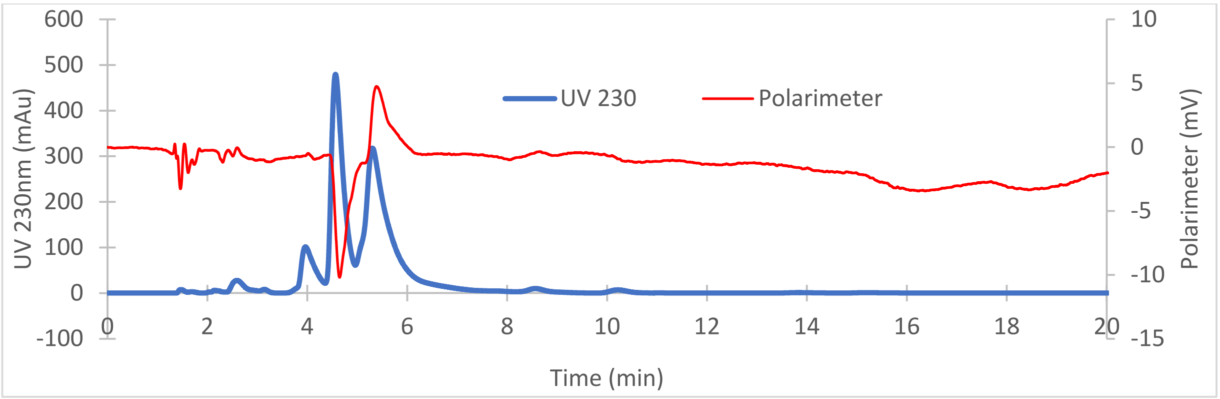

	Retention Time (min)	Area	% Area	Rotation
1	4.56	8185	41.3%	-
2	5.30	11614	58.7%	+

(-)-(4S, 5S)-trans-n-isohumulone and (S)-2-(benzylamino)-1-phenylethanol salt (1:1) (4)

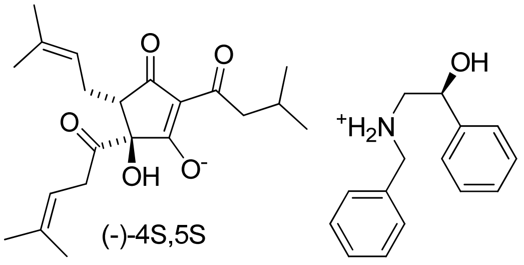

A mixture of 31.4 mg (0.087 mmole) of (S)-(-) trans-*n*-isohumulone and 21.0 mg (0.092 mmole) of (S)-2-(benzylamino)-1-phenylethanol was dissolved in 2 mL ethanol, subsequently filtered and treated with 4 mL of H_2O. The mixture was placed in a freezer overnight. The resultant precipitate was collected by filtration and dried to afford 39 mg (76%) of a crystalline, white solid: mp 137-139 °C.

HPLC (UV 210 nm): 2.00 (63.7%, amine); 12.13 (36%, trans-n-isohumulone)

^{1}H NMR (CDCl$_3$, 300 MHz): δ 9.36 (brs, 2H), 7.29-7.34 (m, 5H), 7.10 – 7.20 (m, 5H), 5.22 (t, 1H, *J* = 6 Hz), 5.13 (t, 1H, *J* = 6.2 Hz), 4.62 (d, 2H, *J* = 9.3 Hz), 4.16 (d, 1H, *J* = 12.3 Hz), 4.03 (d, 1H, *J* = 12.3 Hz), 3.47 (t, 1H, *J* = 11.5 Hz), 3.22-3.31 (m, 2H), 2.98 (dd, 1H, *J* = 19.1 Hz, *J* = 5.0 Hz), 2.75-2.85 (m, 3H), 2.50-2.60 (m 1H), 1.94-2.10 (m, 1H), 1.70 (s, 3H), 1.65 (s, 3H), 1.53 (s, 3H), 1.49 (s, 3H), 0.94 (d, 3H, *J* = 6.5 Hz), 0.93 (d, 3H, *J* = 6.5 Hz).

$[\alpha]_D^{19}$ = +46.3 (EtOH, c = 1.2);

Anal. Calcd for $C_{36}H_{47}NO_6$: C, 73.32; H, 8.03; N, 2.38. Found: C, 73.39; H, 8.13; N, 2.47.

3. X-Ray Data

Seven different chiral amines were investigated as possible candidates for preparation of a salt with trans-iso-n-humulone **2a** that was suitable for x-ray crystallography. While four of the salt combinations provided solids, only (S)-(+)-2-benzylamino-1-phenylethanol gave suitable crystalline solid of x-ray analysis (Table S1)

Table S1. Properties of amine salts with compound **2a**.

Sigma-Aldrich #	CAS #	Name	Properties of isohumulone salt
551953	14467-51-7	(*R*)-(−)-2-tert-Butylamino-1-phenylethanol	Oil
551961	14467-32-4	(*S*)-(+)-2-tert-Butylamino-1-phenylethanol	Oil
551988	107171-75-5	(*R*)-(−)-2-Benzylamino-1-phenylethanol	Mp 91-93
551996	51096-49-2	(*S*)-(+)-2-Benzylamino-1-phenylethanol	Mp 134-136
554472	86906-05-0	(*R*)-(+)-2-Amino-1,1,3-triphenyl-1-propanol	Mp 98 (decomp)
554464	79868-78-3	(*S*)-(−)-2-Amino-1,1,3-triphenyl-1-propanol	Mp 98 (decomp)
	1121-22-8	(1*R*,2*R*)-1,2-cyclohexanediamine	oil

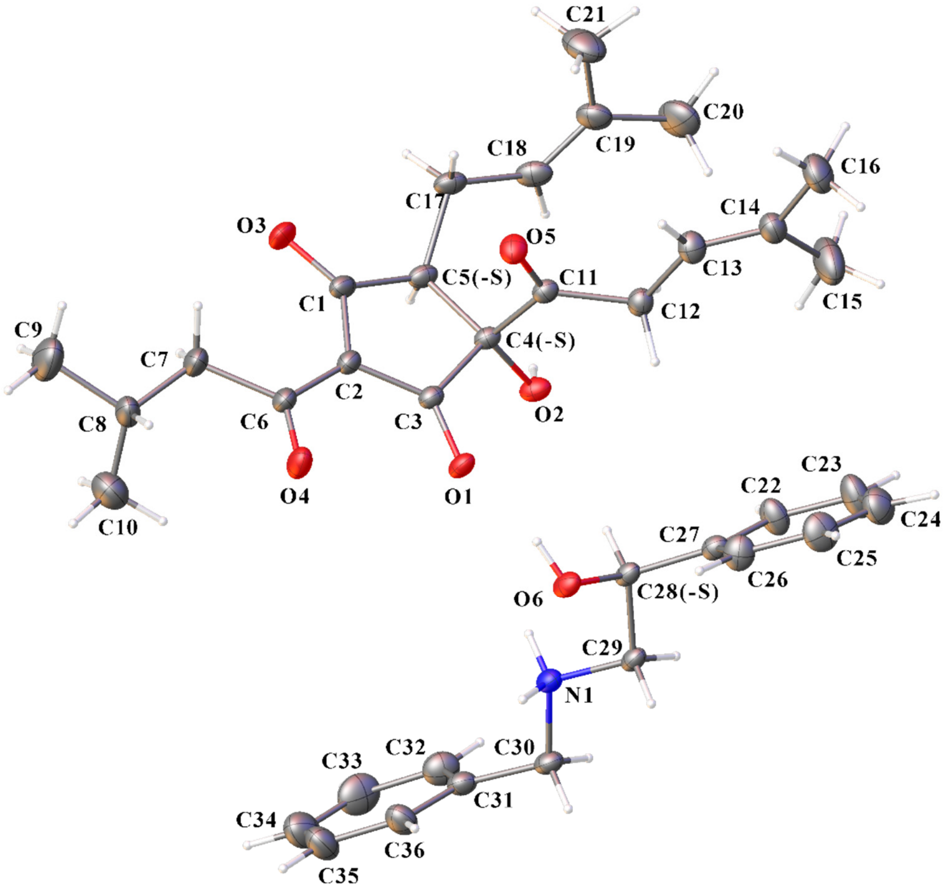

Figure S2: Molecular structure of *trans*-n-isohumulone : (*S*)-2-benzylamino-2-phenylethanol salt (**4**) showing displacement ellipsoids anion drawn at 50% probability level.

Table S2: Crystal Structure refinement details of compound **4**

**Parameters**	**I**
Empirical formula	$C_{36}H_{47}NO_6$
Moiety formula	$C_{29}H_{29}O_5$, $C_{15}H_{18}NO$
Formula weight	589.74
Temperature (K)	276.0
Wavelength (Å)	1.54178
Crystal system, space group	Orthorhombic, $P2_12_12_1$
Unit cell dimensions	
a (Å)	5.85130(10)
b (Å)	19.2363(4)
c (Å)	30.2947(7)

α (˚)	90
β (˚)	90
γ (˚)	90
Volume (Å^3)	3409.89(12)
Z, Calculated density (Mg/m^3)	4, 1.149
Absorption coefficient, μ (mm^{-1})	0.618
F(000)	1272.0
Crystal size (mm)	0.3 × 0.25 × 0.08
Theta range for data collection (˚)	2.72 to 67.9
Limiting indices	-7 ≤ h ≤ 6, -23 ≤ k ≤ 22, -36 ≤ l ≤ 36
Reflections collected	38937
Independent reflections	6181 [R_{int} = 0.0901, R_{sigma} = 0.0478]
Data / restraints / parameters	6181 / 18 / 402
Goodness-of-fit on F^2	1.033
Final R indices [I>2sigma(I)]	R_1 = 0.0636, wR_2 = 0.1844
R indices (all data)	R_1 = 0.0786, wR_2 = 0.1977
Largest diff. peak and hole (eÅ^{-3})	0.44 and -0.26
Flack Parameter	0.14(9)

Single crystal data collection and refinement.

Plate shaped or flaky type crystals of (**4**) [*trans*-n-isohumulone:(*S*)-2-benzylamino-2-phenylethanol salt] were obtained from crystallization process, suitable size of 0.3x0.25x0.08mm was chosen for X-Ray diffraction experiment. Intensity data of (**4**) was collected using Bruker D8 Venture Photon II Diffractometer equipped with micro-focus sealed tube of wavelength 1.5418 Å (Cu-source). Correct unit-cell parameters were determined by collecting data from 60 frames in three different crystallographic zones followed by data collection with exposure time of 10s per frame and sacn width of 0.5˚. Data reduction, scaling and multi-scan absroption correction were carried out using SAINT-plus and SADABS in APEX6 software [4]. The crystal structure was solved by direct methods procedure using SHELXS-2018 program and refined by Full-matrix least squares procedure on F^2 using SHELXL-2018 [5] program. The intensity data was checked for missing symmetry elements or inversion twin with PLATON program [6]. The atomic displacement parameters (ADP) of atoms C8 to C10 were made similar using suitable similarity restraints followed by suitable rigid bond restraints were used, which alows the adjacent atom to move in the bond direction and refined. Hydrogen atoms were placed in geometrically idealised positions and constrained to ride on their parent atoms with C-H distances in the range 0.93-0.97(Å). Isotropic thermal parameters (Ueq) were fixed such that they were Uiso(H) = 1.5Uequ(C) for methyl and (for aromatic CH) with Uiso(H) = 1.2Uequ(C) methylene group respectively. Whereas, the hydroxyl hydrogen atoms are restrained to a O-H distance of 0.85(1) (Å) with Uiso(H) = 1.5Uequ(C). The molecular graphics images of the molecules were drawn using Olex2 [7] software. The crystal structure refinement details are summarized in Table 1. Finalized crystal structure of (**4**) was deposited in Cambridge Structural Database and their reference Id is CCDC #2573601.

4. References

1) Dong, M. W. *Modern HPLC for Practicing Scientists*. Wiley-Interscience: New Jersey, USA, **2006**.

2) Hamper, B. C.; Gallow, B.; Giovine, G.; Smith, T. Continuous-flow photochemical isomerization of humulones to isohumulones. *Molecules*, **2025,** *30,* 1002.

3) Hamper, B. C.; Campbell, H. J.; Luo, R.; Murphy, M.; Gleason, P.; Smith, T.; Jagan, R. Selective synthesis of deuterated cis- and trans-isohumulones and trans-isohumulinones. *Synthesis,* **2024**, *56*, 3206-3214.

4) Bruker, APEX3, SAINT-Plus and SADABS (2016). Bruker AXS Inc., Madison, Wisconsin, USA.

5) G. M. Sheldrick, "Crystal structure refinement with SHELXL". *Acta Crystallographica C*. (**2015**) 71, 3-8.

6. Spek, A. L. Structure validation in chemical crystallography. *Acta Crystallographica Section D*, (**2009**), 65, 148-155

7. Dolomanov, O. V., Bourhis, L. J., Gildea, R. J., Howard, J. A. K., Puschmann, H. “OLEX2: A complete structure solution, refinement and analysis program”. *Journal of Applied Crystallography*, **(2009),** *42(2),* 339-341.

4. NMR Data

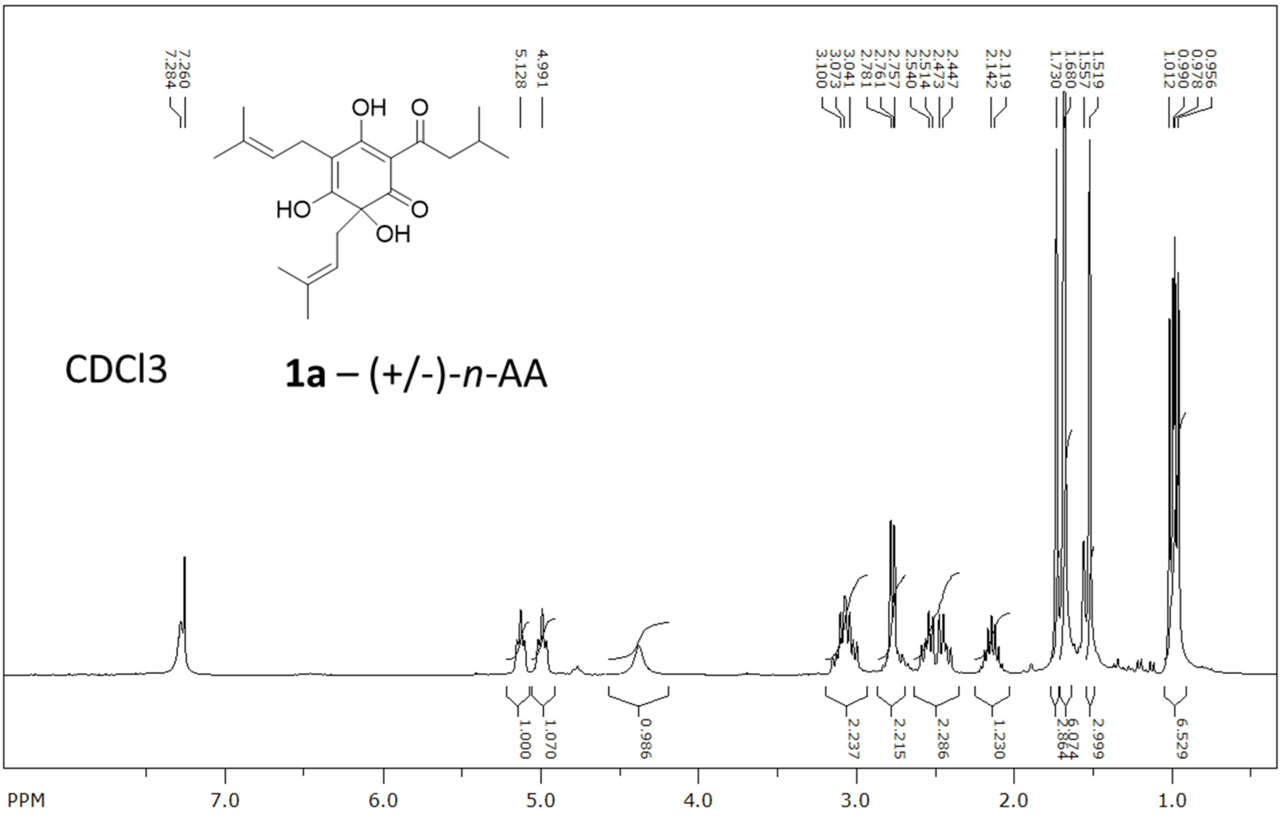

file: H:\BCH3-181-A\10\fid expt: <zg30>
transmitter freq.: 300.131853 MHz
time domain size: 65536 points
width: 6172.84 Hz = 20.5671 ppm = 0.094190 Hz/pt
number of scans: 16

freq. of 0 ppm: 300.130015 MHz
processed size: 32768 complex points
LB: 0.300 GF: 0.0000

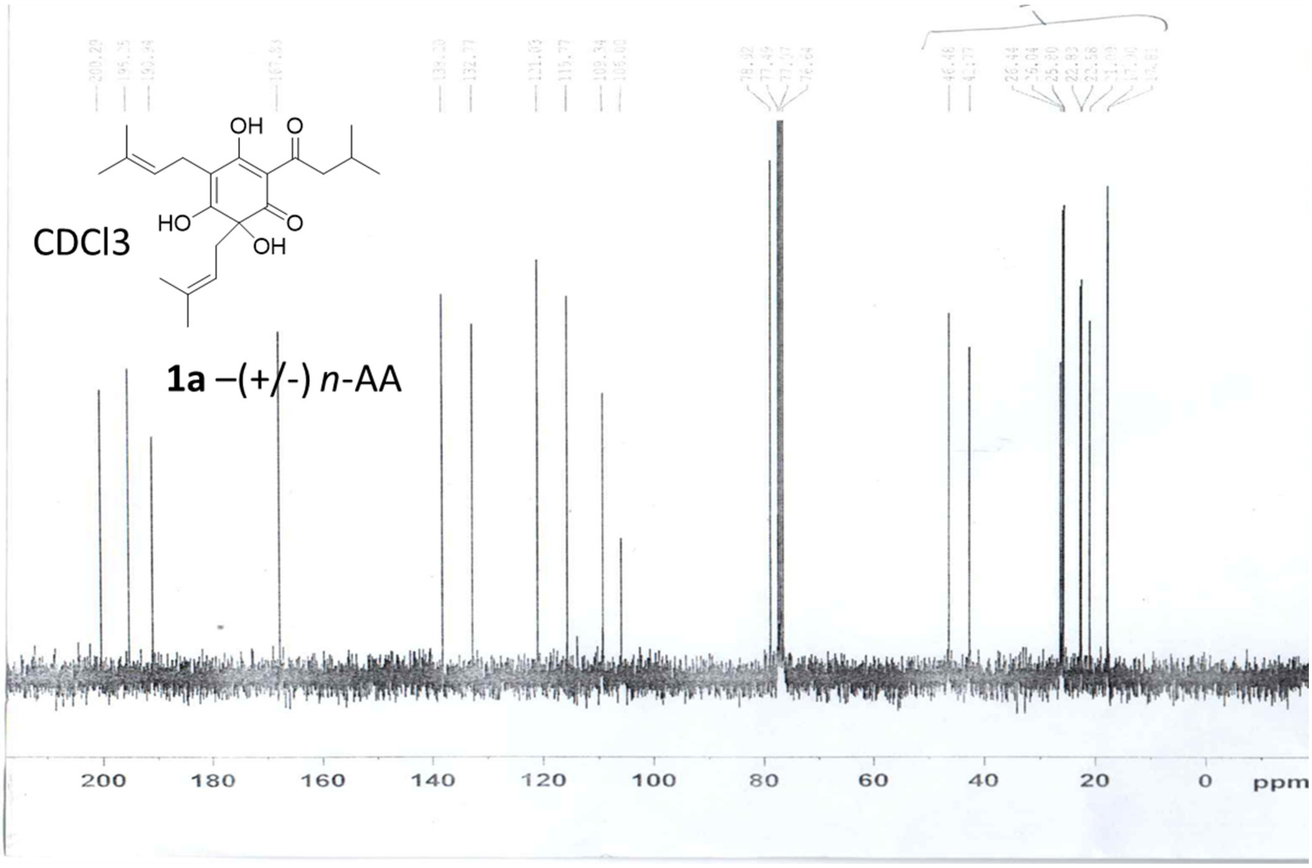
CDCl3
OH
O
HO
O
OH
1a –(+/-) n-AA
200 180 160 140 120 100 80 60 40 20 0 ppm

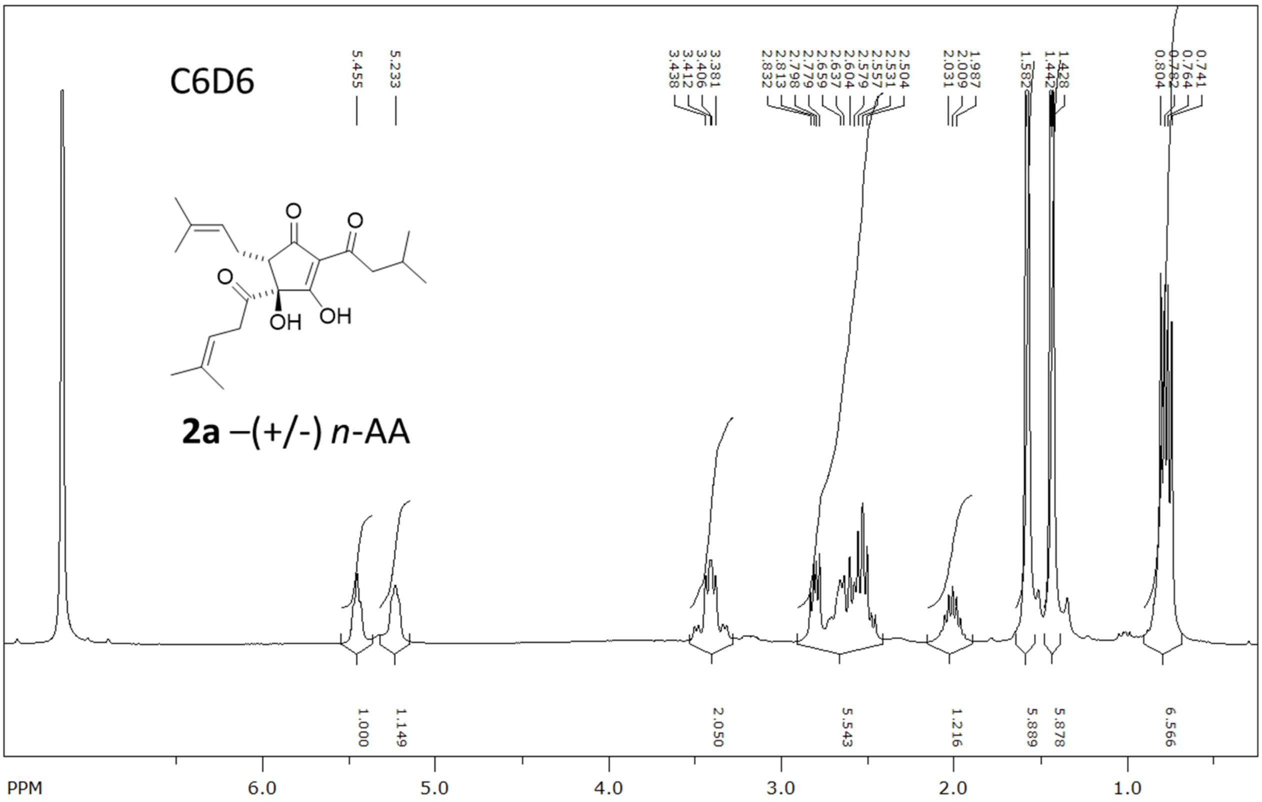

file: H:\bch3-188\10\fid expt: <zg30>
transmitter freq.: 300.131351 MHz
time domain size: 65536 points
width: 3306.88 Hz = 11.0181 ppm = 0.050459 Hz/pt
number of scans: 32

freq. of 0 ppm: 300.130032 MHz
processed size: 32768 complex points
LB: 1.000 GF: 0.0000

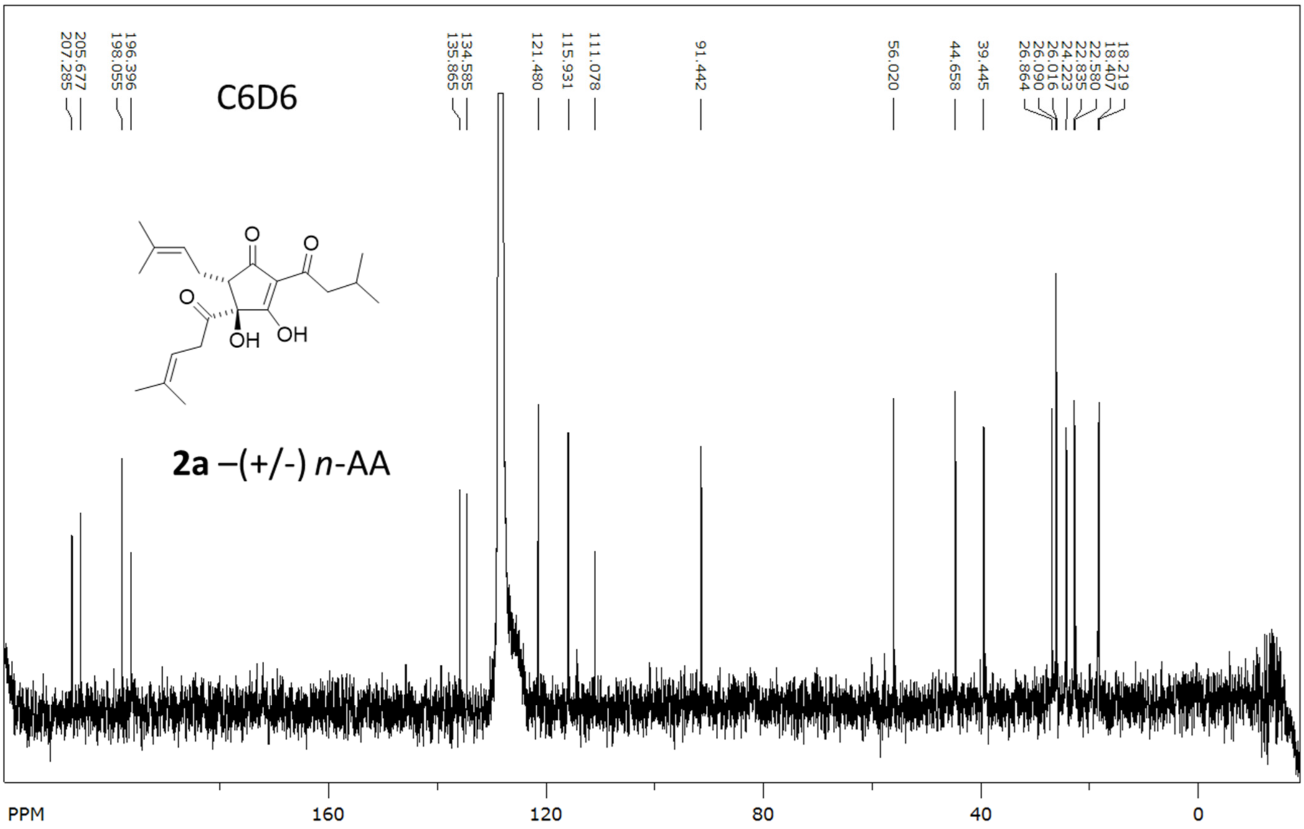

file: H:\bch3-188\11\fid expt: <zgpg30>
transmitter freq.: 75.475295 MHz
time domain size: 65536 points
width: 17985.61 Hz = 238.2980 ppm = 0.274439 Hz/pt
number of scans: 10240

freq. of 0 ppm: 75.467705 MHz
processed size: 32768 complex points
LB: 2.000 GF: 0.0000

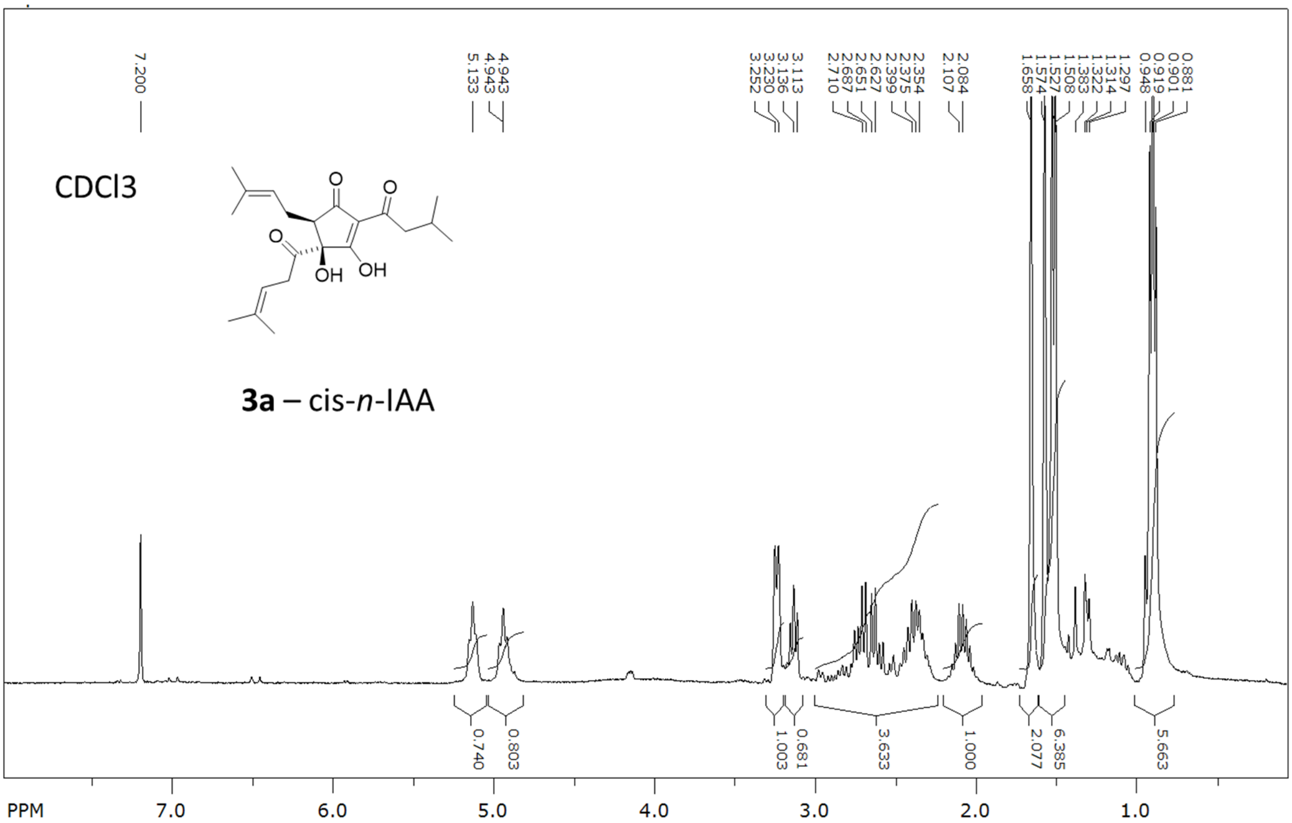

file: G:\BCH3-040-20\fid expt: <zg30>
transmitter freq.: 300.131853 MHz
time domain size: 65536 points
width: 6172.84 Hz = 20.5671 ppm = 0.094190 Hz/pt
number of scans: 16

freq. of 0 ppm: 300.130033 MHz
processed size: 32768 complex points
LB: 0.300 GF: 0.0000

CDCl3

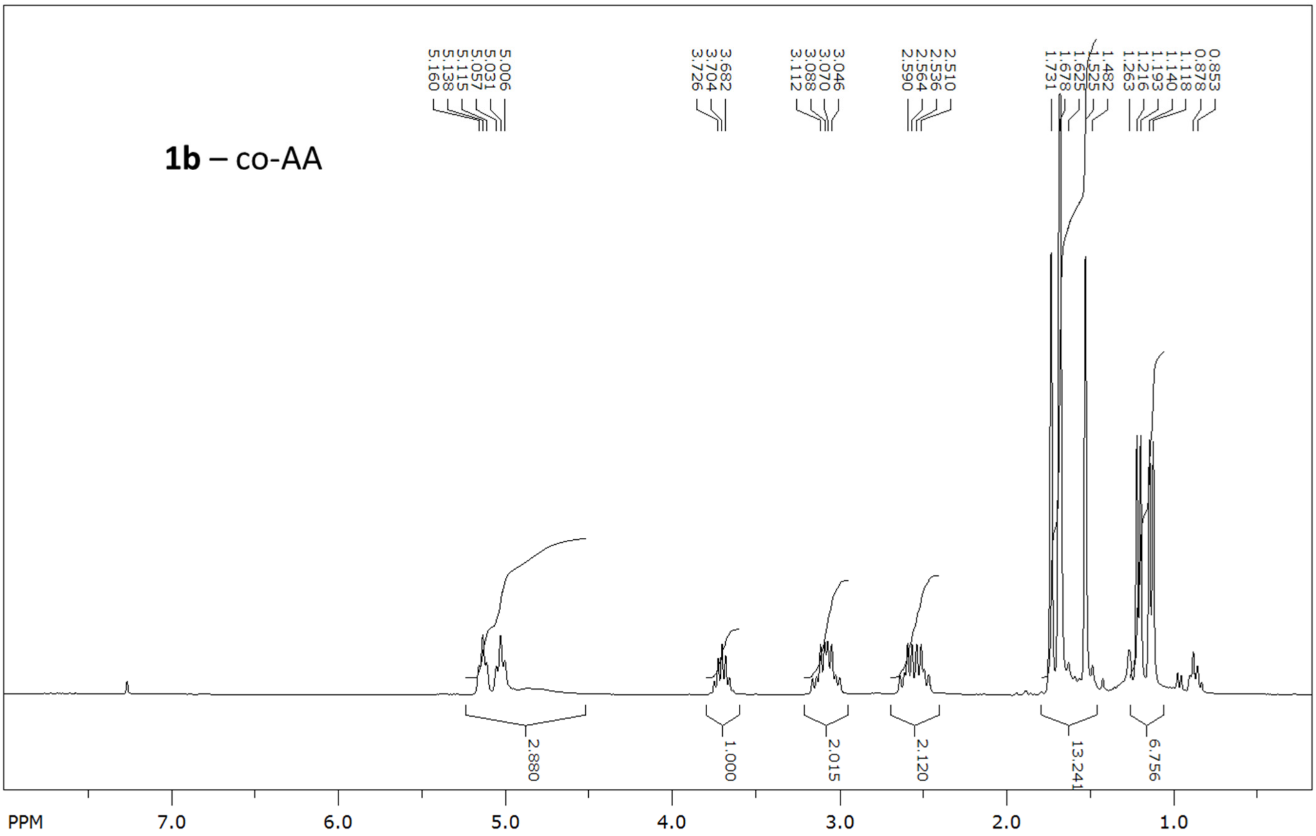

file: ...\PG-079-cohumulone-NMR data\10\fid expt: <zg30>
transmitter freq.: 300.131853 MHz
time domain size: 65536 points
width: 6172.84 Hz = 20.5671 ppm = 0.094190 Hz/pt
number of scans: 16

freq. of 0 ppm: 300.130012 MHz
processed size: 32768 complex points
LB: 0.300 GF: 0.0000

CDCl3

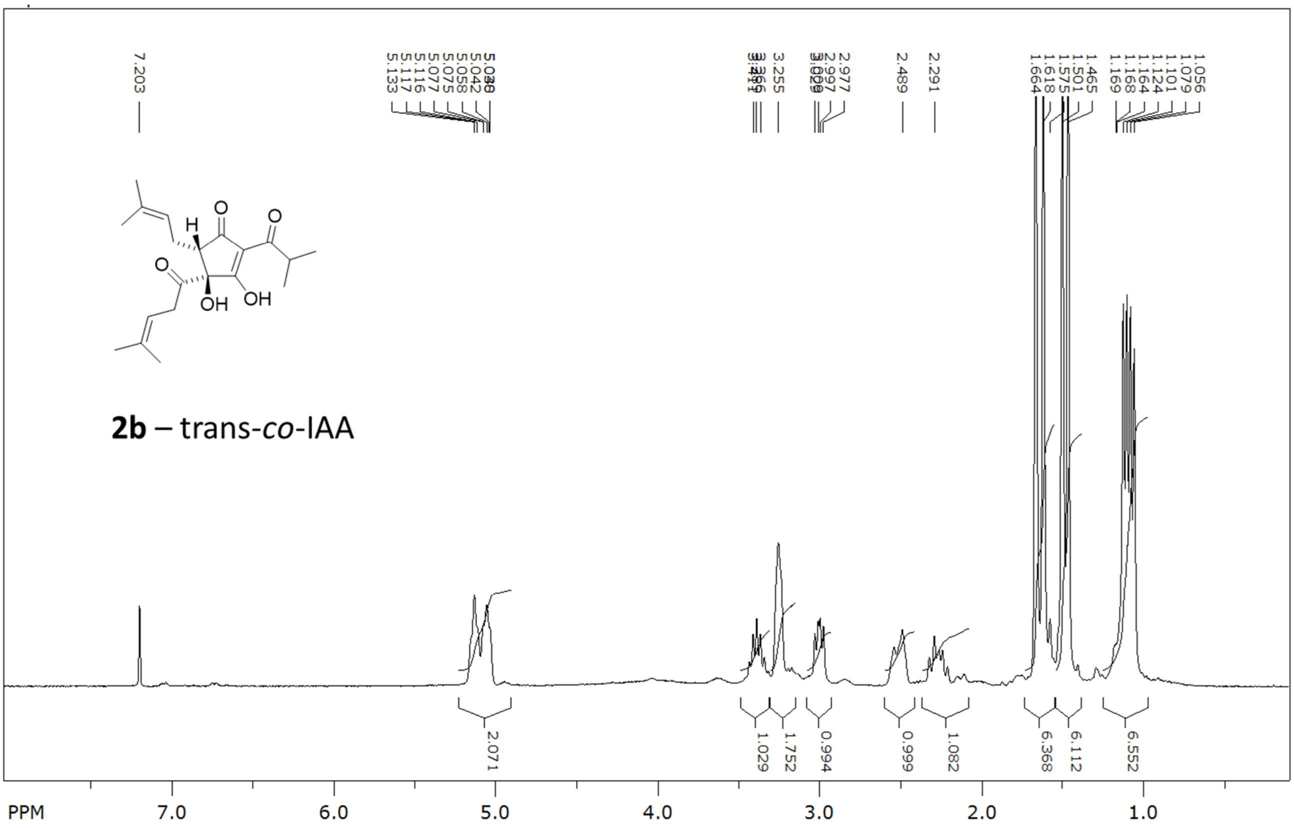

file: G:\bch3-099-2\1\fid expt: <zg30>
transmitter freq.: 300.131853 MHz
time domain size: 65536 points
width: 6172.84 Hz = 20.5671 ppm = 0.094190 Hz/pt
number of scans: 16

freq. of 0 ppm: 300.130033 MHz
processed size: 32768 complex points
LB: 0.300 GF: 0.0000

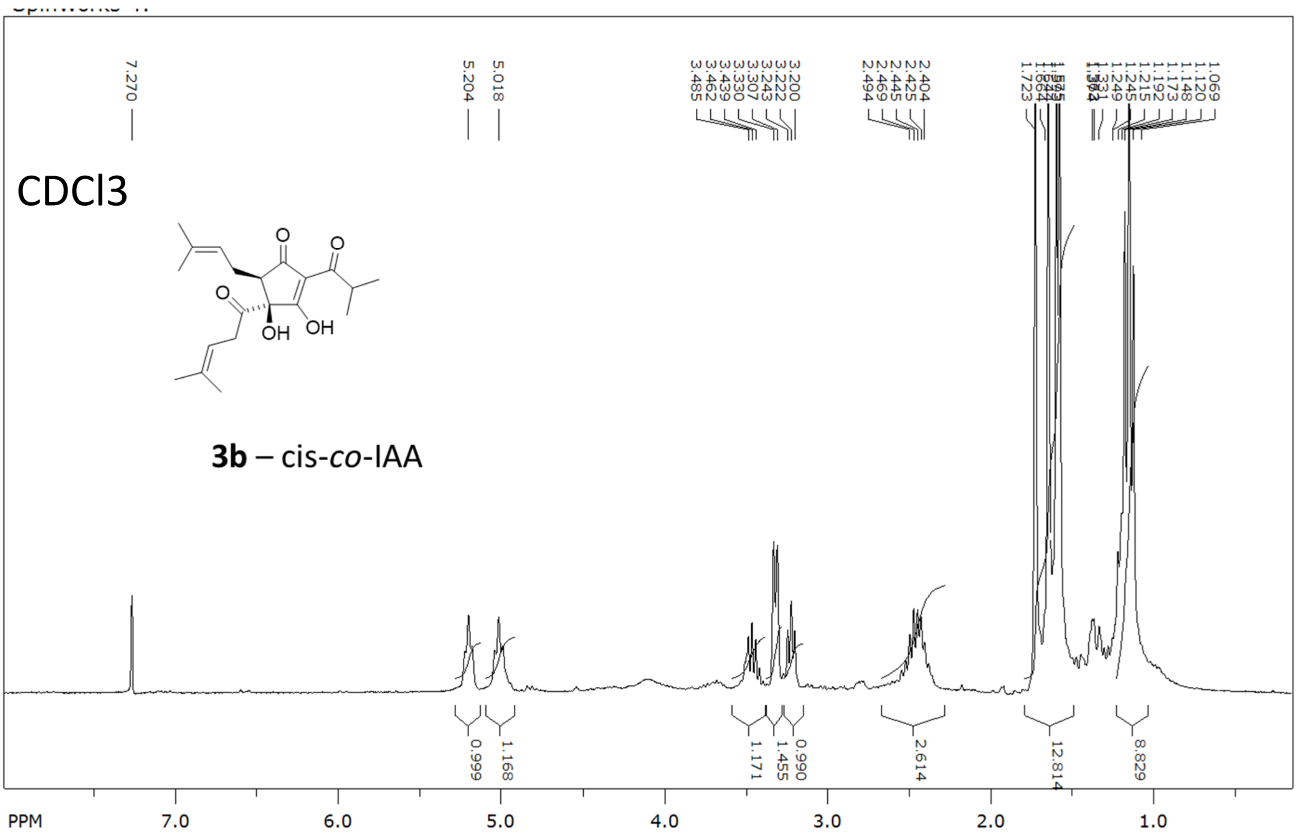

file: G:\BCH3-069-4\10\fid expt: <zg30>
transmitter freq.: 300.131853 MHz
time domain size: 65536 points
width: 6172.84 Hz = 20.5671 ppm = 0.094190 Hz/pt
number of scans: 16

freq. of 0 ppm: 300.130012 MHz
processed size: 32768 complex points
LB: 0.300 GF: 0.0000

CDCl3

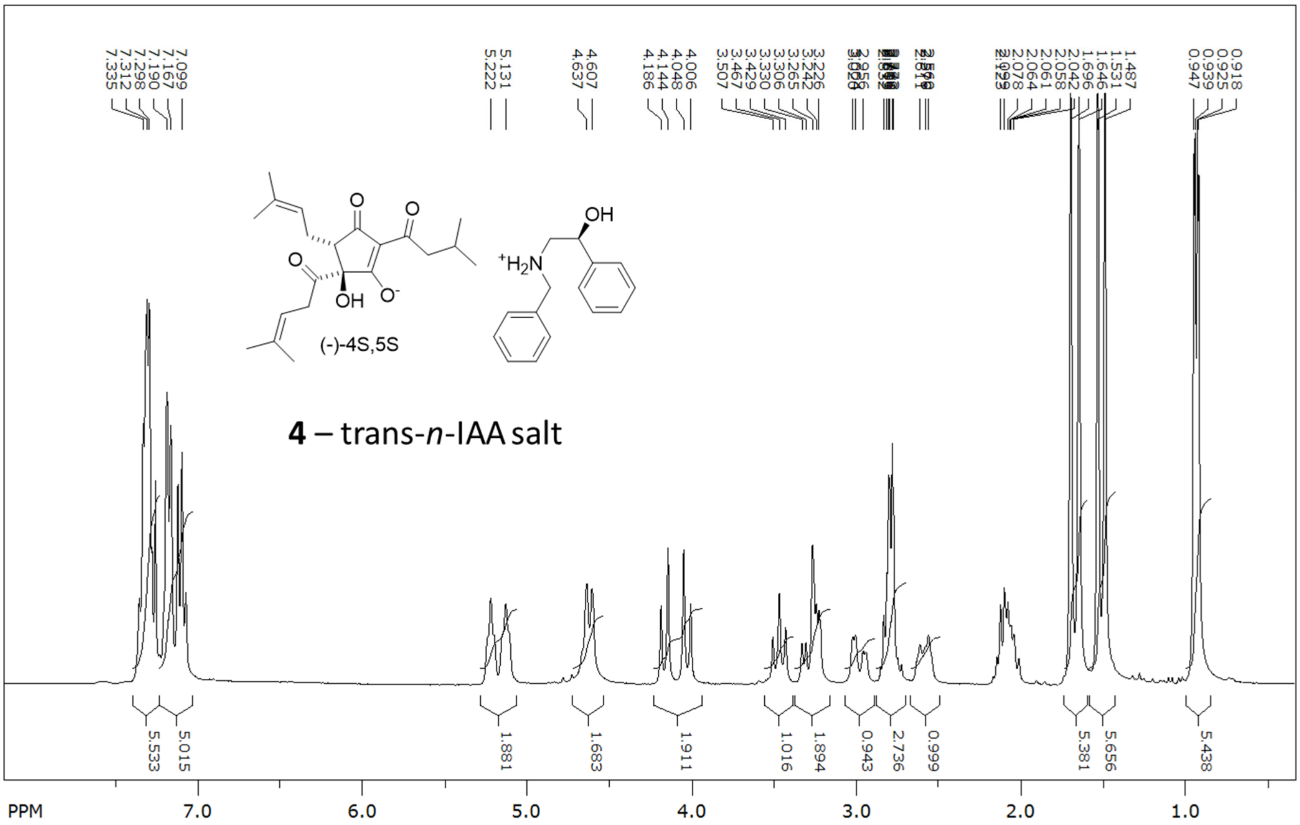

file: ...3\data\bh\nmr\Jan28-2026-bh\10\fid expt: <zg30>
transmitter freq.: 300.131853 MHz
time domain size: 65536 points
width: 6172.84 Hz = 20.5671 ppm = 0.094190 Hz/pt
number of scans: 16

freq. of 0 ppm: 300.130015 MHz
processed size: 32768 complex points
LB: 0.300 GF: 0.0000
